# Mitochondria-targeting nanomedicines with autophagy inhibitor to enhance cancer photothermal-chemotherapy

**DOI:** 10.1093/rb/rbae141

**Published:** 2025-01-06

**Authors:** Shuqi Chen, Wenxia Gao, Shuhua Chang, Bin He, Congbo Zhang, Miaochang Liu, Xueting Ye

**Affiliations:** School of Pharmacy, Chengdu University, Chengdu 610106, China; School of Pharmacy, Chengdu University, Chengdu 610106, China; National Engineering Research Center for Biomaterials, College of Biomedical Engineering, Sichuan University, Chengdu 610064, China; National Engineering Research Center for Biomaterials, College of Biomedical Engineering, Sichuan University, Chengdu 610064, China; College of Chemistry and Materials Engineering, Wenzhou University, Wenzhou 325027, China; College of Chemistry and Materials Engineering, Wenzhou University, Wenzhou 325027, China; Department of Urology, First Affiliated Hospital of Wenzhou Medical University, Wenzhou 325000, China

**Keywords:** mitochondria-targeting, autophagy inhibition, multi-functional nanoparticles, photothermal-chemotherapy

## Abstract

In this article, we fabricated nanomedicines with mitochondrial targeting function and autophagy inhibitor for enhancing cancer photothermal-chemotherapy. The nanoparticles were fabricated with gold nanoparticles (AuNPs) as cores and amphiphilic dextran with (3-carboxypropyl) triphenyl phosphorus bromide and β-cyclodextrin (β-CD) modification (TPP-DCD) as shells; the chemotherapeutic doxorubicin (DOX) and autophagy inhibitor chloroquine (CQ) were encapsulated in the nanoparticles. The TPP-DCD was synthesized via the immobilization of 2-aminoethanethiol modified β-CD and (3-carboxypropyl) triphenylphosphonium bromide on dextran to receive coordination interaction with AuNPs and mitochondria targeting. The size, morphology and properties of the Au@DOX/CQ@TPP-DCD nanoparticles were studied. The nanomedicines efficiently targeted cellular mitochondria to produce reactive oxygen species and photothermal effect under NIR irradiation. The released DOX and CQ could not only kill tumor cells directly, but also inhibit the autophagy of cancer cells to enhance therapeutic effects. Both *in vitro* and *in vivo* anticancer activities of the nanomedicines were investigated in detail. The *in vivo* imaging demonstrated that the Au@DOX/CQ@TPP-DCD nanomedicines exhibited efficient targeting, accumulation and retention in tumor-bearing mice. The apoptosis of cancer cells and tumor suppression were greatly accelerated with the addition of 808 nm NIR irradiation. The Au@DOX/CQ@TPP-DCD nanomedicine exhibited significant synergistic therapy, as 75% of tumors in mice disappeared. The Au@DOX/CQ@TPP-DCD nanoparticle is a promising nanomedicine for cancer therapy with synergistic effects.

## Graphical Abstract

**Figure:**
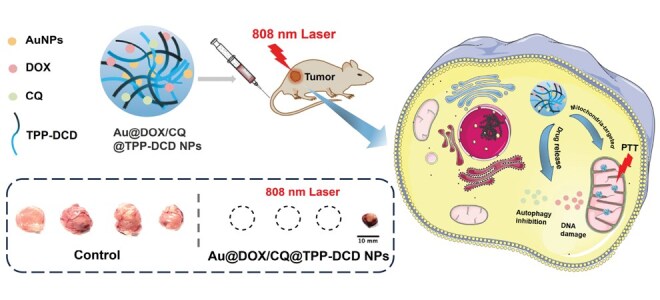

## Introduction

Cancers pose significant challenges and result in the deaths of more than 10 million people each year [1]. New strategies, including photodynamic therapy [2, 3], photothermal therapy (PTT) [4–6], sonodynamic therapy [7, 8], chemodynamic therapy [9–12] and immunotherapy [13], have been developed for treatment.

PTT destroys cell membranes and/or denatures proteins at high temperature using photothermal materials, such as organic dyes or nanomaterials, to convert light energy into thermal energy [14]. PTT also generates excessive reactive oxygen species (ROS) such as hydroxyl radicals, hydrogen peroxide superoxide and singlet oxygen species to destruct DNA, proteins and lipids, and then induces the apoptosis of cancer cells [15]. The generated heating and ROS are controllable precisely via the intensity and duration of light as well as photothermal material concentrations [16]. Near-infrared light (NIR) is utilized to induce PTT with the irradiation of photothermal agents (PTAs) due to its deep tissue penetration [17].

Many organic and inorganic PTAs are investigated. Au nanoparticles (AuNPs) are one of the most important PTAs in tumor PTT [18]. The AuNPs include nanorods [19, 20], nanoshells [21], cages [22, 23], nanorings [24, 25] and chiral nanoparticles [26, 27]. AuNPs coated with proteins, peptides, aptamers and small molecules are usually used to enhance tumor eradication and/or photothermal conversion [28–34]. Multiply functionalized AuNPs with stimuli-sensitivity and organelles/cells/tissues targeting are reported [35–43]. Mitochondria are organelles known as ‘the energy factories’ of cells, responsible for producing ROS. The destruction of mitochondria cuts off the 'energy supply' of cells, triggering cellular apoptosis [44]. Triphenyl phosphate and its derivatives are preferentially targeting mitochondria via endocytosis to decrease the potential of mitochondrial membrane and induce cell death [45–47].

Autophagy is an intracellular defense behavior. The tumor cells initiate autophagy pathway to resist the damage caused by light and heat and lead to ineffective tumor inhibition [48]. Cancer cells degrade and circulate misfolded proteins and disordered organelles to reconstruct the cellular microenvironment via autophagy [49]. In PTT, the damaged proteins and organelles are cleaved by autophagy to repair and reverse cell damage, leading to incomplete cell necrosis [50, 51]. To overcome the resistance, stringent conditions (such as high temperatures and extended irradiation times) are applied in PTT to avoid the damage of normal tissues. The initiation of autophagy is involved in multiple pathways [52–54]. It was reported that low PTT temperature only killed tumor cells efficiently, but did not initiate autophagy [55, 56]. The nanoparticles loaded with autophagy inhibitor hydroxychloroquine exhibited efficient anticancer activity under mild PTT temperature [57].

**Figure 1.**
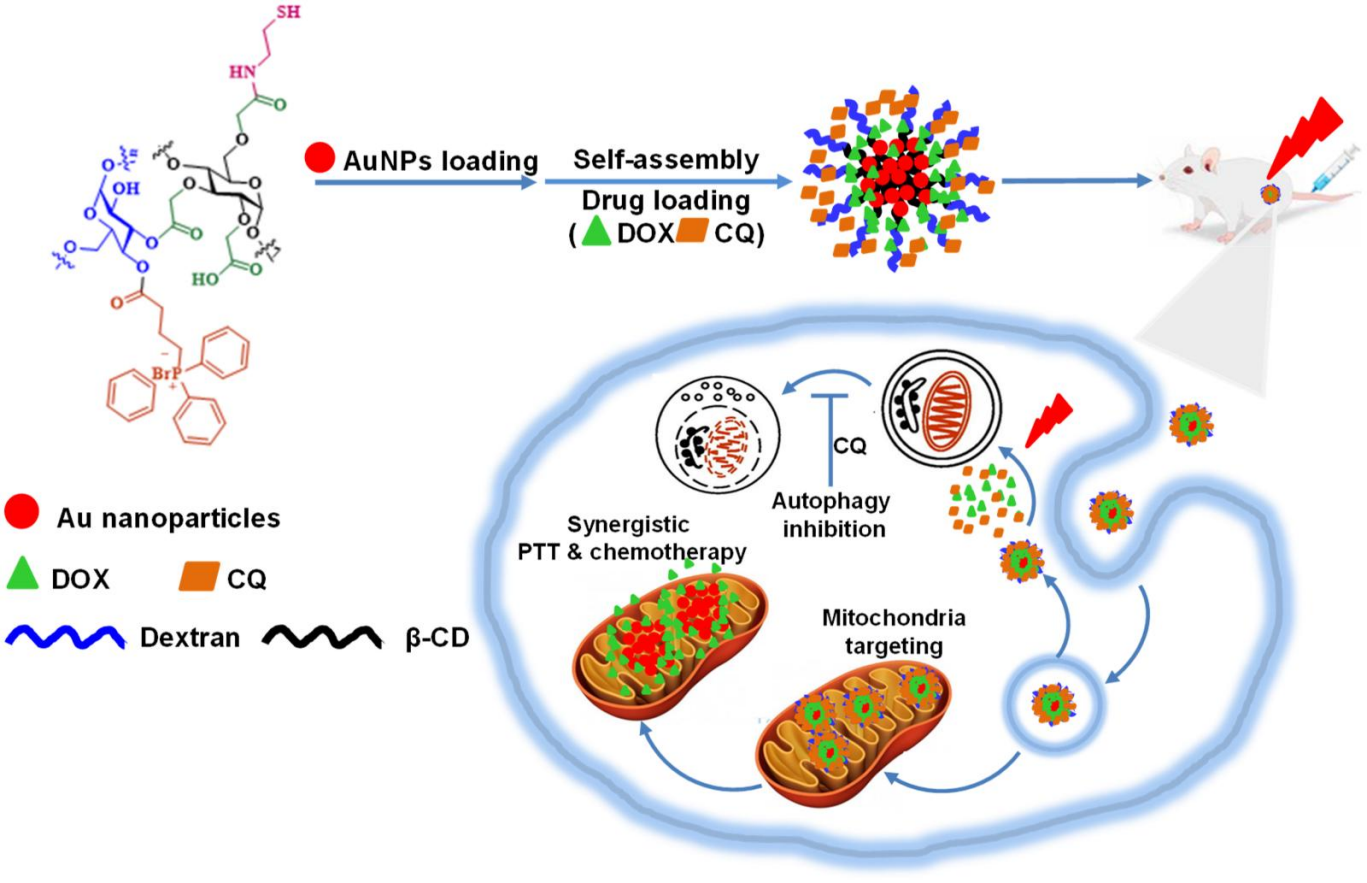
The schematic demonstration of mitochondria targeting nanomedicines to enhance synergistic cancer therapy via autophagy inhibition.

In this article, we developed nanomedicines with mitochondria-targeting and autophagy-inhibition functions to enhance cancer therapy. The autophagy inhibitor chloroquine (CQ) and chemotherapeutic doxorubicin (DOX) were co-loaded in nanoparticles with AuNPs as cores and (3-carboxypropyl) triphenyl phosphorus bromide/β-cyclodextrin (β-CD)-modified dextran (TPP-DCD) as shells (Figure 1). The efficient cancer treatment resulted not only from the chemotherapeutic effects of DOX and the PTT of AuNPs under NIR irradiation but also from the autophagy inhibition of CQ.

The Experimental section is presented in the Supplementary Material.

## Results and discussion

### Characterizations of TPP-DCD

**Figure 2.**
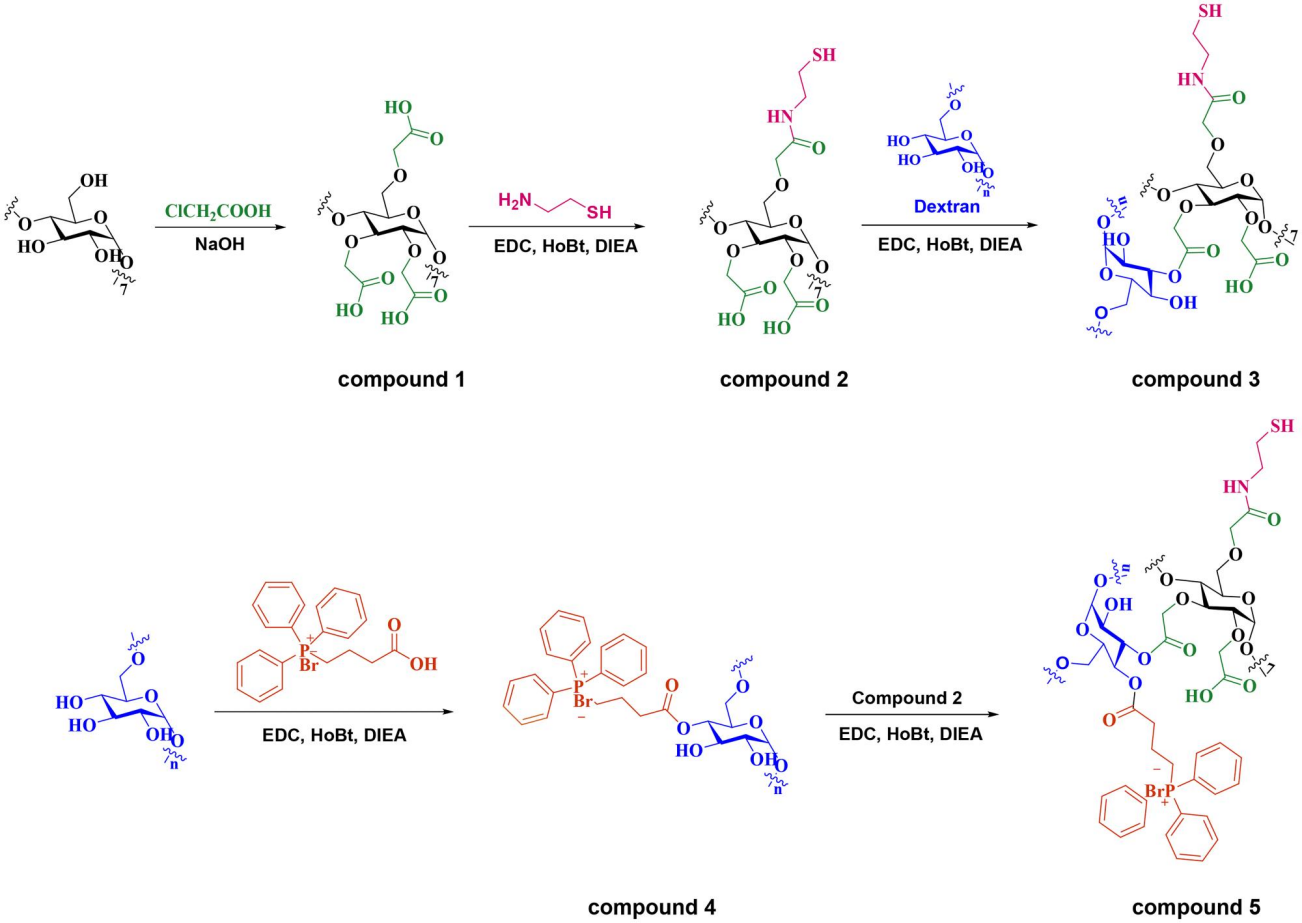
The synthesis of TPP-DCD.

The mitochondria targeting polymer TPP-DCD was synthesized as shown in Figure 2. The polysaccharide dextran was used as the matrix to immobilize mitochondria targeting unit TPP and β-CD for drug loading improvement. The β-CD was carboxylated to prepare compound 1 (β-CD-COOH). The typical proton signals of β-CD appeared at δ=5.74, 5.68, 4.82, 4.47, 3.565–3.55 and 3.3 ppm, which were attributed to the protons in 2-OH, 3-OH, 6-OH and 1-6 H in cyclodextrin (Figure S1). The ^1^H NMR spectrum of β-CD-COOH is shown in Figure S2; the resonance peak at 5.08 ppm was assigned to the characteristic peak of methylene in -O-CH_2_-COOH and the signal at 4.30 ppm was the proton of methylene in 8′-O-CH_2_-COOH and 9′-O-CH_2_-COOH. In addition, the shape change and integrity decrease of the peaks at 5.68 ppm (3-OH) and 5.74 ppm (2-OH) revealed the replacement of -OH on β-CD, implying the successful synthesis of β-CD-COOH (compound 1).

**Figure 3.**
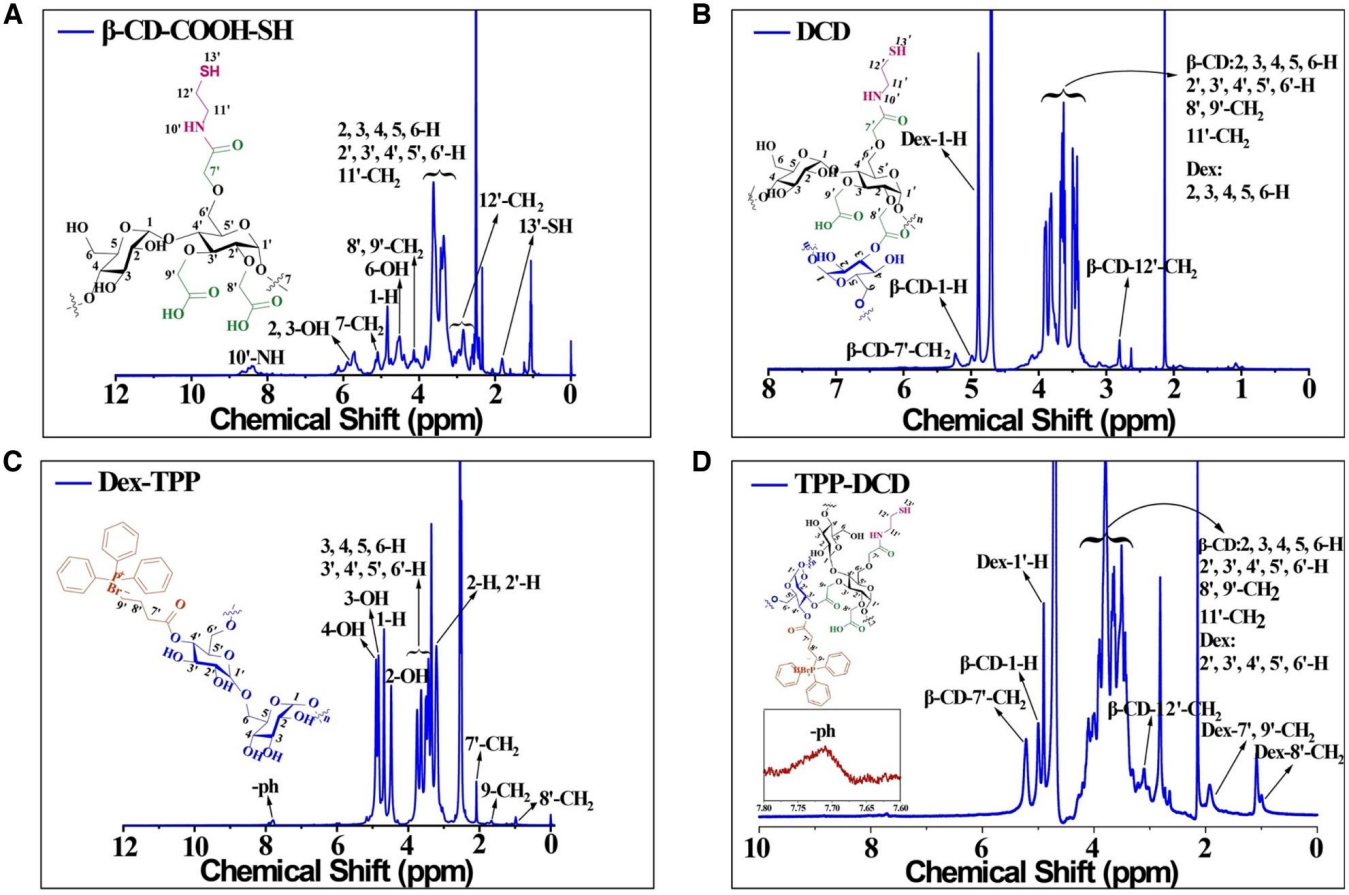
^1^H NMR Spectra of the compounds, (**A**) compound 2, β-CD-COOH-SH; (**B**) Compound 3, DCD; (**C**) Compound 4, Dex-TPP; (**D**) Compound 5, TPP-DCD.

Compound 1 was further modified with 2-aminoethanethiol to prepare compound 2 (β-CD-COOH-SH). The ^1^H NMR spectrum is shown in Figure 3A. The characteristic peak at δ=8.41 ppm was attributed to the proton of –CO–NH; the peak at 4.41 ppm was assigned to the methylene in 8′-O-CH_2_-COOH and 9′-O-CH_2_-COOH. The peak at 3.81 ppm was attributed to the proton of methylene close to imine in –NH–CH_2_–CH_2_–SH, the peak at 2.84 ppm was assigned to the sulfhydryl group in –NH–CH_2_–CH_2_–SH and the peak at 1.81 ppm was attributed to the proton of –SH. In the FT-IR spectra (Figure S3), the wave number at 3395 cm^−1^ (N–H) and 1588 cm^−1^ (C(O)-NH) represented the stretching vibration peaks of the amide bonds.

The β-CD-COOH-SH was further bonded to dextran to prepare compound 3 (DCD, Dex-β-CD-COOH-SH). The characteristic peaks of dextran were 1-H at 4.68 ppm, 4-OH at 4.899 ppm, 3-OH at 4.82 ppm, 2-OH at 4.47 ppm. The peaks of 3.34–3.74 ppm were the protons of 3, 4, 5 and 6-H; the peak at 3.21 ppm was attributed to the proton of 2-H (Figure S4). The ^1^H NMR spectrum of compound 3 (DCD) was shown in Figure 3B. The peak at 5.32 ppm was attributed to the methylene of –O–CH_2_–COOH in β-CD, the peak at 5.08 ppm was the proton of 1-H in β-CD, the peak at 4.98 ppm was 1-H in Dextran and the peak at 2.89 ppm was the proton of methylene close to sulfhydryl groups in β-CD –NH–CH_2_–CH_2_–SH. In the FT-IR spectrum (Figure S5), the peak at 1596 cm^−1^ represented the expansion and contraction vibration of the amide bond (C(O)-NH) in DCD.

The mitochondria targeting group TPP was immobilized on dextran to prepare compound 4 (Dex-TPP). The ^1^H NMR spectrum of Dex-TPP was shown in Figure 3C. The peak at 7.74 ppm was assigned to the proton of benzene ring, the peak at 2.04 ppm was attributed to the proton of methylene group in –CH_2_–COO–, the signal at 1.61 ppm was the methylene group of –P^+^Br–CH_2_ and the peak at 0.94 ppm was the methylene group far away from ester group –CH_2_–CH_2_–COO. The FT-IR spectra were available in Figure S6; the peak at 1567 cm^−1^ was the skeleton vibration peak of benzene ring, the peak at 1700 cm^−1^ represented the telescopic vibration of C=O and the peak at 1439 cm^−1^ represented the telescopic vibration peak of –(CH_2_)_3_. These results illustrated the successful preparation of Dex-TPP.

The mitochondria targeting polymer compound 5 (Dex-TPP-β-CD-COOH-SH, TPP-DCD) was synthesized. The ^1^H NMR spectrum of compound 5 is shown in Figure 3D. The signal at 7.80 ppm was the proton of benzene ring, the peak at 5.31 ppm was the methylene group of –O–CH_2_–COOH on β-CD, the signal at 5.08 ppm was the proton of 1-H in β-CD, the peak at 4.99 ppm was the proton of 1-H in dextran, the peak at 2.90 ppm was the proton of the methylene group near the sulfhydryl group in –NH–CH_2_–CH_2_–SH on β-CD, the signal at 2.73 ppm was the proton of the methylene group in –CH_2_–COO in Dex-TPP and the peak at 2.01 ppm was the proton of the methylene group in –P^+^Br–CH_2_ of Dex-TPP, and the peak at 1.18 ppm was the proton of the methylene group far away from ester group in –CH_2_–CH_2_–COO in Dex-TPP. In the FT-IR spectrum of Dex-TPP, the stretching vibration peak at 1596 cm^−1^ was attributed to the amide bond (C(O)-NH) in β-CD-COOH-SH, the bending vibration of benzene ring appeared at 913 cm^−1^ (Figure S5).

Thermogravimetric study was carried out to further confirm the synthesis of the polymer. As shown in Figures S7 and S8, the weight loss of β-CD-COOH-SH decreased to 48% during 230–330°C, and that of β-CD was 85%. The weight loss of β-CD-COOH-SH slowed down to 37% as the temperature increased from 330°C to 570°C, which was attributed to the thermal decomposition of carboxyl and hydroxyl groups. The weight loss of DCD was 50% between 210°C and 360°C, gradually decreasing to 22% as the temperature rose from 300°C to 500°C. The weight loss of TPP-DCD was 67% when the temperature increased from 180°C to 340°C, and the complete decomposition temperature was as high as 700°C.

### Characterizations of nanoparticles

**Figure 4.**
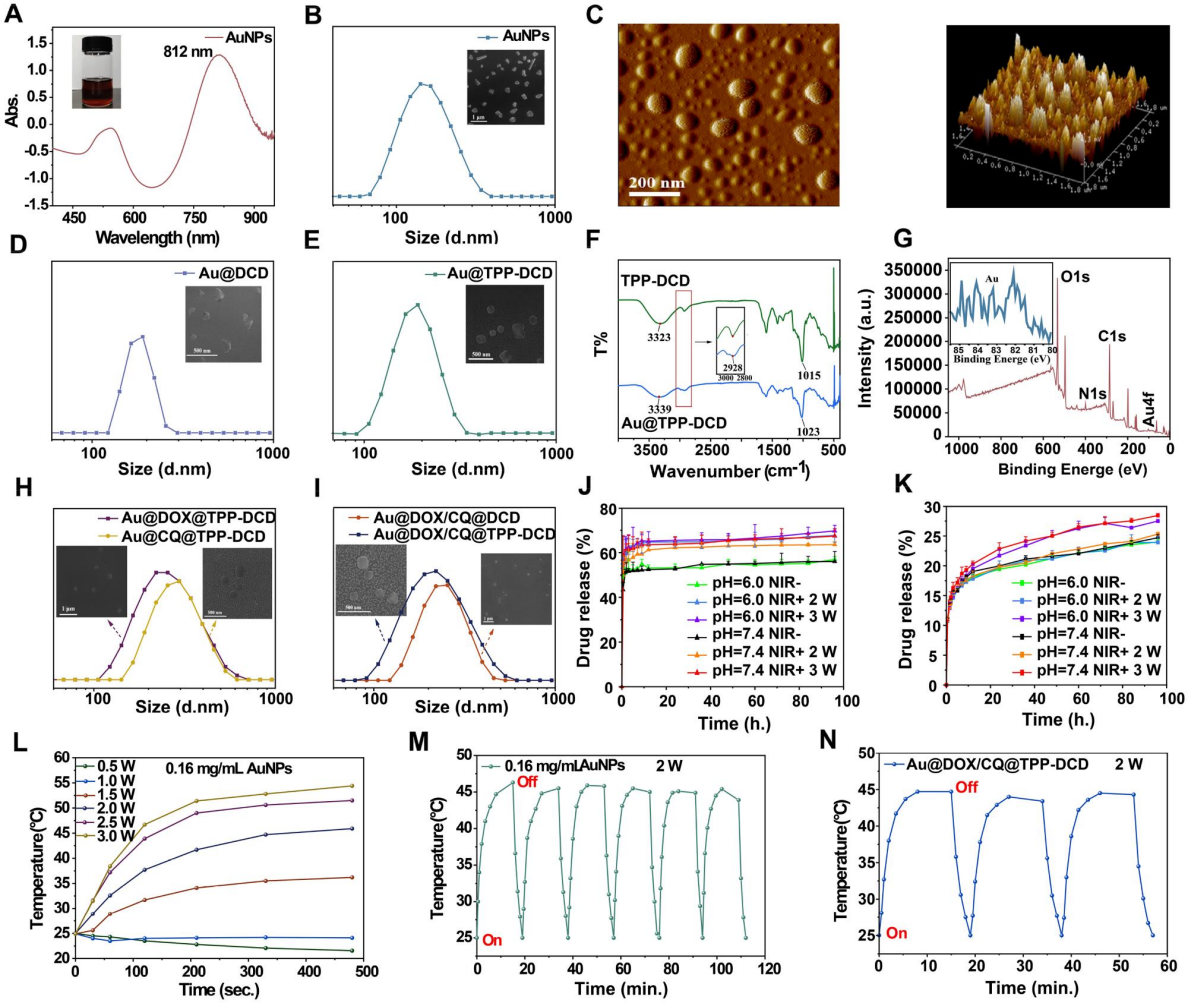
Morphology and photothermal effects of nanoparticles, (**A**) UV spectrum of AuNPs, (**B**) size distribution and SEM image of AuNPs, (**C**) 2D and 3D atomic force microscopy images of AuNPs, (**D**) size distribution and SEM image of Au@DCD, (**E**) size distribution and SEM image of Au@TPP-DCD, (**F**) FT-IR spectra of Au@DCD and Au@TPP-DCD, (**G**) XPS spectrum of Au@TPP-DCD, (**H**) size distribution and SEM images of Au@DOX@TPP-DCD and Au@CQ@TPP-DCD, (**I**) size distribution and SEM images of Au@DOX/CQ@DCD and Au@DOX/CQ@TPP-DCD, (**J**) release curve of chloroquine *in vitro*, (**K**) release curve of doxorubicin *in vitro*, (**L**) the photothermal curves of AuNPs under different NIR irradiations (808 nm), (**M**) the cyclic photothermal curves of AuNPs under NIR irradiation (808 nm, 2 W), (**N**) the cyclic photothermal curves of Au@DOX/CQ@TPP-DCD under NIR irradiation (808 nm, 2 W).

The AuNPs were involved as the cores in the mitochondria targeting nanoparticles. As shown in Figure 4A, AuNPs were prepared and dispersed well in water with a UV maximum absorption peak of 812 nm. The AuNPs appeared rod-shaped in the SEM image (Figure 4B). The surface topography of the AuNPs was further characterized with atomic force microscopy, and the length of the nanorods ranged from 200 to 400 nm (Figure 4C).

The AuNPs were coated with amphiphilic polymer DCD to form Au@DCD nanoparticles (Figure 4D); the nanoparticle size was around 200 nm with a rough surface. The AuNPs coated with TPP-DCD exhibited excellent dispersibility, as shown in Figure 4E. As the hydrophobicity of TPP-DCD was higher than that of DCD, the TPP-DCD coated AuNPs were more conducive to assemble into regular nanoparticles. The particle size of both the Au@DCD and Au@TPP-DCD nanoparticles was around 200 nm, which was consistent with the results in SEM images (Figure 4D and E). The particle size of DCD or TPP-DCD coated AuNPs increased compared to the original AuNPs.

The FT-IR spectra of TPP-DCD and TPP-DCD coated AuNPs nanoparticles were measured. The O–H expansion vibration peak at 3387 cm^−1^, the C–H expansion vibration peak at 2928 cm^−1^ and the C–O expansion vibration peak of CH_2_OH at 1015 cm^−1^ in TPP-DCD are available in Figure 4F. To Au@TPP-DCD, the O–H vibration peak was shifted from 3387 to 3339 cm^−1^; the peak shape was broadened and the peak intensity was weakened. The C–H telescopic vibration peak at 2928 cm^−1^ was broadened too. The C–O expansion vibration peak at 1015 cm^−1^ was shifted to 1023 cm^−1^, the peak type was widened and the intensity became weaker. Figure 4G showed the XPS spectrum of Au@TPP-DCD, the signals of C, O, H, N and Au were found. Two peaks at 83.5 and 87.2 eV in the spectra were assigned to AuNPs. The concentration of Au colloids in Au@TPP-DCD nanoparticles was estimated to be about 0.06%.

Anticancer drug DOX and autophagy inhibitor CQ were encapsulated in the Au@DCD and Au@TPP-DCD nanoparticles, which were marked as Au@DOX@TPP-DCD, Au@CQ@TPP-DCD, Au@DOX/CQ@TPP-DCD and Au@DOX/CQ@DCD. The zeta potential of the four drug-loaded nanoparticles was negative (Figure S9). The drug loading content and encapsulation efficiency of the nanoparticles are shown in Table S1. The size distribution and SEM images of the four drug-loaded nanoparticles are shown in Figure 4H and 4I. All the nanoparticles were round and spherical with good dispersibility; the particle size was between 200 and 300 nm. In the release profiles, the PBS with different pH values showed less effect on the cumulative release (Figure 4J). However, the release of DOX improved with increasing power of irradiation. The cumulative release of DOX in PBS (pH 6.0) under the NIR irradiation was about 28% with 3 W (808 nm) and 23% with 2 W (808 nm). The release rate of CQ in PBS (pH 6.0) was slightly higher than that in PBS (pH 7.4), either with or without NIR irradiation (Figure 4J). The cumulative release of CQ reached 69.69% in PBS (pH 6.0) with NIR irradiation (808 nm, 2 W) within 5 h.

The photothermal conversion of AuNPs was carried out under NIR (808 nm) irradiation at different powers. As shown in Figure 4L, the temperature of AuNPs (Au concentration, 0.16 mg/mL) did not increase under NIR irradiation at the powers of 0.5 and 1.0 W; the temperature of AuNPs increased from 25°C to 35°C when the NIR power was 1.5 W. When the NIR power increased to 2 W, 2.5 W, 3.0 W, the temperature could reach 37.8°C, 43.9°C and 46.7°C in 120 s. The temperature further increased to 41.7°C, 49.0°C and 51.4°C in 210 s, and to 45.9°C, 51.5°C and 54.4°C in 480 s, respectively. Higher temperatures were achieved with an increase in NIR power (Figure 4L and Figure S10).

The stability of the photothermal conversion effect of AuNPs was further studied. Under NIR irradiation (808 nm, 2 W), the heating curve of AuNPs was shown in Figure 4M. The AuNPs were heated from 25°C to 46°C for a 15-min irradiation; the irradiation was stopped and the temperature of AuNPs returned to 25°C. This process was regarded as one cycle. After six cycles, the temperature was not changed, revealing the good photothermal stability of AuNPs. The Au@DOX/CQ@TPP-DCD nanoparticles could also maintain the stable photothermal conversion as shown in Figure 4N and Figure S11.

### *In vitro* cytotoxicity assessment

**Figure 5.**
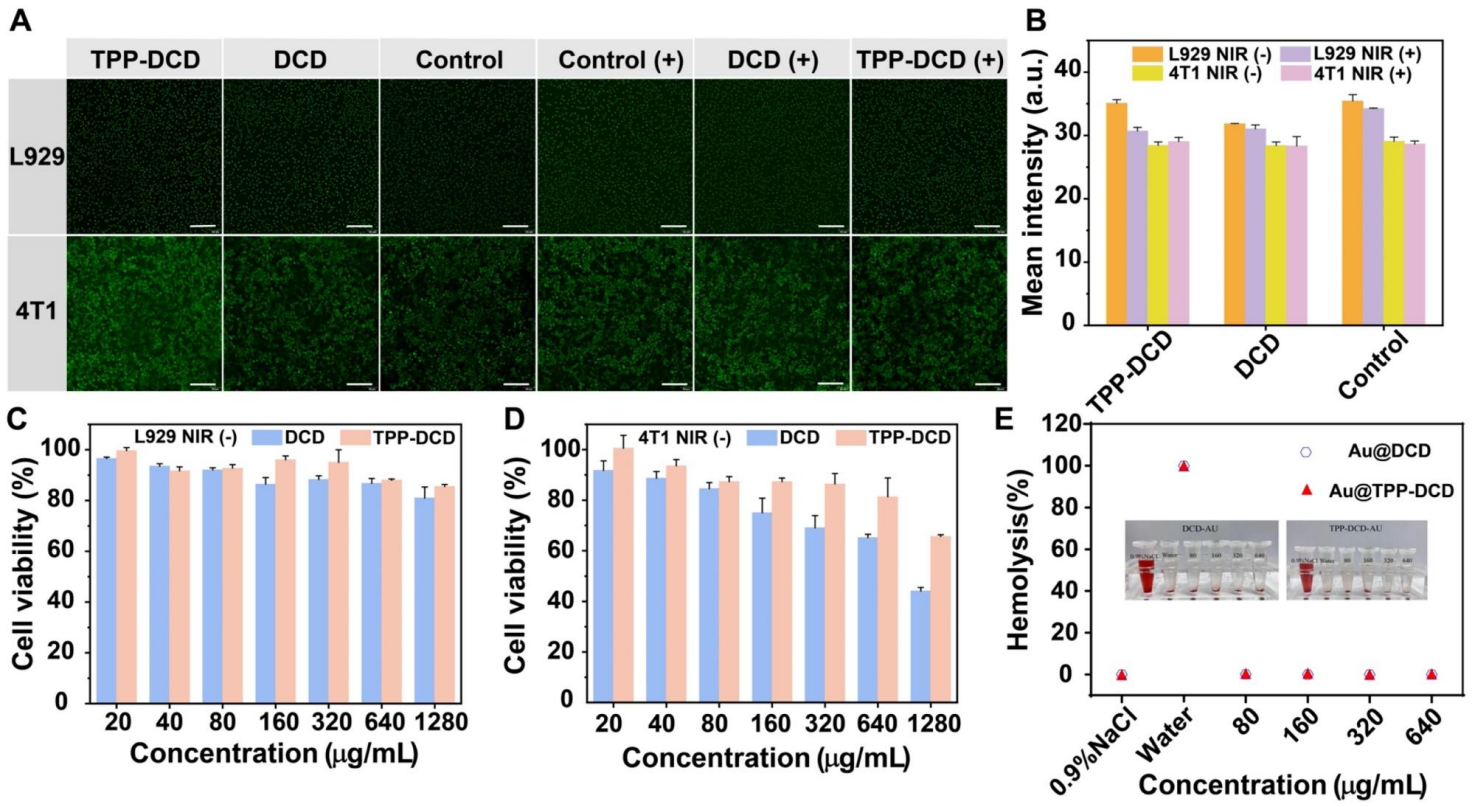
(**A**) Confocal fluorescent images of live/dead L929 cells and 4T1 cells stained using calcein-AO (green, living cells) and EB (red, dead cells) after addition of DCD/TPP-DCD (80 μg/mL) with or without laser irradiation. The NIR irradiation intensity was 1.0 W/cm^2^, and the irradiation duration was 3 min, (scale bar = 100 μm); (**B**) statistical analysis of the intracellular fluorescence intensities in (**A**); cytotoxicity profiles of L929 cells (**C**) and 4T1 cells (**D**) after incubating with DCD and TPP-DCD for 24 h; (**E**) percent hemolysis of red cell suspension incubated with DCD and TPP-DCD at 37°C for 1 h. Water was used as a positive control of hemolysis, while saline solution was a negative control.

The cytocompatibility and hemocompatibility evaluation of the polymer carriers was performed on L929 cells and 4T1 cells using the AO/EB staining method. Acridine orange emitted bright green fluorescence once passed through living cells with intact cell membranes and embedded itself in nuclear DNA (Figure 5A). Both the DCD and TPP-DCD groups, at a concentration of 80 μg/mL, exhibited bright green fluorescence, whether under NIR irradiation (808 nm, 1 W/cm^2^, 3 min) or not. At the same time, there was almost no red fluorescence of dead cells in these groups, confirming the excellent biocompatibility of polymers. Semi-quantitative calculation of the fluorescence images exhibited non-toxicity of the two groups (Figure 5B).

MTT assays were carried out to detect the cell viability of L929 cells and 4T1 cells after co-culturing with DCD and TPP-DCD for 24 h, with concentrations from 20 to 1280 μg/mL [58, 59]. As shown in Figure 5C, when the concentration was less than 80 μg/mL, the L929 cell viability exceeded 90%. When the concentrations of DCD and TP-DCD were lower than 640 μg/mL, the cell viabilities of both DCD and TPP-DCD exceeded 85%. Even when the concentration of DCD and TPP-DCD reached 1280 μg/mL, the cell viabilities of DCD and TPP-DCD were 81.04% and 85.58%, respectively.

As shown in Figure 5D, when the TPP-DCD concentration was less than 320 μg/mL, the cell survival rate was more than 85%. Even when the concentration was increased to 640 μg/mL, the cell survival rate was still 81.41%, but when the concentration was further increased to 1280 μg/mL, the cell survival rate decreased to 65.71%. This indicated that TPP-DCD exhibited negligible cytotoxicity to 4T1 cells in a certain concentration range. For DCD, it exhibited dose-dependent cytotoxicity to 4T1 cells. Only when the DCD concentration was less than 80 μg/mL, the cell survival rate was higher than 80%; with the concentration increasing, the cell survival rate of DCD gradually decreased. When the concentration increased to 1280 μg/mL, the cell survival rate of DCD was only 44.15%.

In addition, as shown in Figure 5E, when the polymer concentration ranged from 80 to 640 μg/mL, the hemolytic activity of DCD and TPP-DCD to red blood cells (RBC) was negligible, indicating that the two carrier materials were hemocompatible.

### *In vitro* anticancer activity

**Figure 6.**
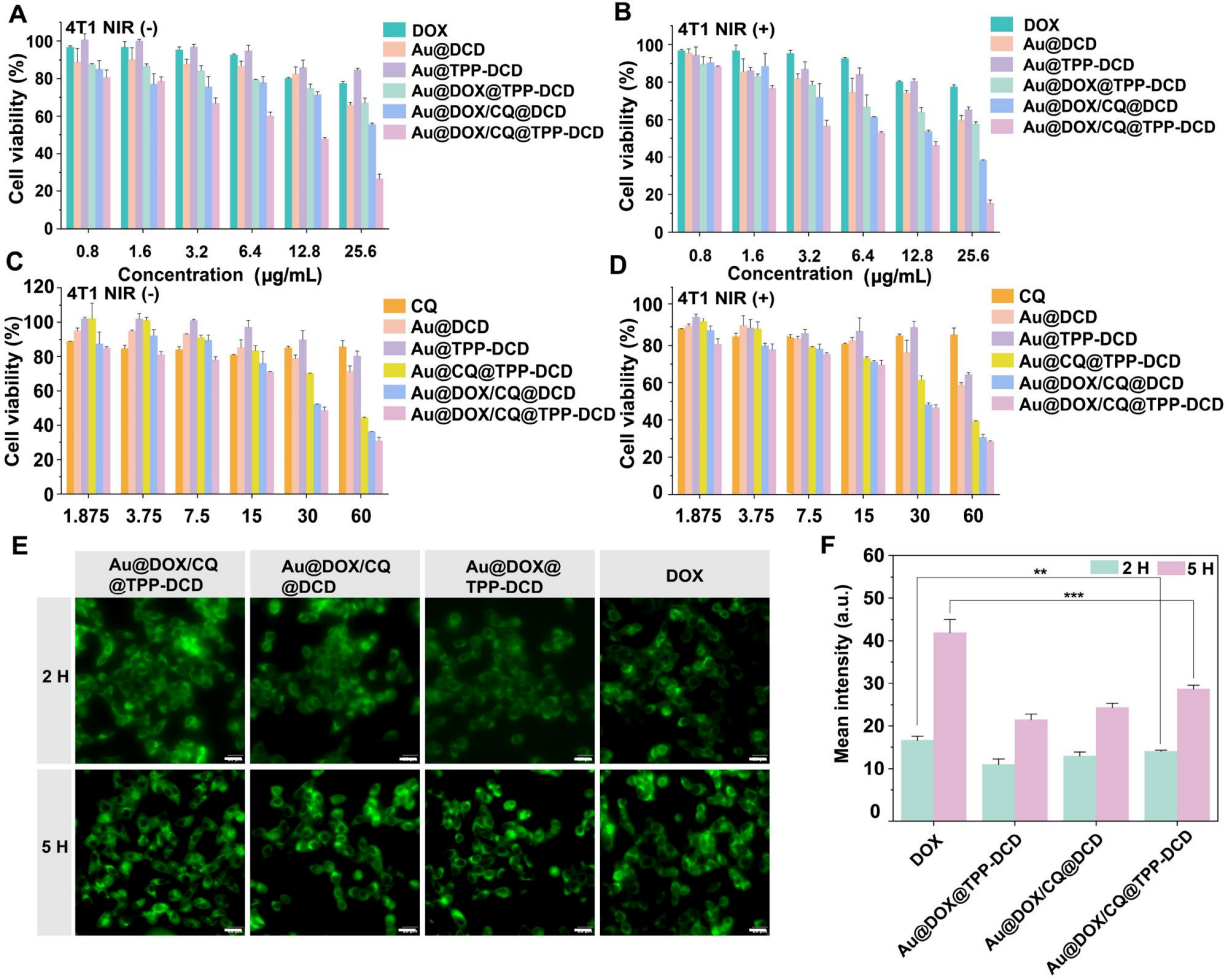
*In vitro* therapeutic performance and cellular uptake. (**A**) Cell viability of 4T1 cells after incubated with different concentrations of DOX, Au@DCD, Au@TPP-DCD, Au@DOX@TPP-DCD, Au@DOX/CQ@DCD, Au@DOX/CQ@TPP-DCD for 24 h, the concentrations of DOX were varied. (**B**) Cell viability of 4T1 cells incubated with different concentrations of DOX, Au@DCD, Au@TPP-DCD, Au@DOX@TPP-DCD, Au@DOX/CQ@DCD and Au@DOX/CQ@TPP-DCD for 5 h and irradiated with 808 nm laser for 3 min followed by continued incubation for 19 h, the concentrations of DOX were varied. (**C**) Cell viability of 4T1 cells after incubation with different concentrations of CQ, Au@DCD, Au@TPP-DCD, Au@CQ@TPP-DCD, Au@DOX/CQ@DCD, Au@DOX/CQ@TPP-DCD for 24 h, the concentrations of CQ were varied. (**D**) Cell viability of 4T1 cells incubated with different concentrations of DOX, DCD-Au, Au@TPP-DCD, Au@DOX/CQ@DCD, Au@DOX/CQ@DCD and Au@DOX/CQ@TPP-DCD for 5 h and irradiated with 808 nm laser for 3 min followed by continued incubation for 19 h, the concentrations of CQ were varied. (**E**) Cellular uptake, (scale bar = 20 μm). (**F**) Statistical analysis of the intracellular fluorescence intensities in (E). Statistical significance was performed by one-way ANOVA test. **: *P* < 0.01; ***: *P* < 0.0001.

The *in vitro* tumor inhibition activities of six treatment groups to 4T1 cells, including DOX, Au@DCD, Au@TPP-DCD, Au@DOX@TPP-DCD, Au@DOX/CQ@DCD and Au@DOX/CQ@TPP-DCD, were studied, as shown in Figure 6A and B. The survival rate of 4T1 cells treated with Au@TPP-DCD group without NIR (808 nm) laser irradiation was above 85% in all concentration range. In fact, the cell viabilities of the Au@DCD and Au@TPP-DCD groups in Figure 6A–D were the cytotoxicity of the nanoparticles, as no therapeutics were encapsulated in the two nanoparticles, and the concentrations of nanoparticles were equivalent to the corresponding carrier concentrations of the drug-loaded samples. The cell survival rates of both free DOX and Au@DOX@TPP-DCD groups showed a downward trend with increasing DOX concentration. The cell survival rates were 92.63% and 79.37% when the DOX concentration was 6.4 μg/mL. The mitochondria targeting contributed to the increased cell apoptosis of Au@DOX@TPP-DCD. When the nanocarrier was co-loaded with DOX and autophagy inhibitor CQ, the cell survival rate of Au@DOX/CQ@TPP-DCD group decreased to 60.06% at the same DOX concentration of 6.4 μg/mL, indicating the synergistic therapeutic effect of Au@DOX/CQ@TPP-DCD. The autophagy inhibitor CQ could promote the anticancer activity of DOX.

Nanoparticle groups with the same DOX concentration were studied under NIR irradiation to evaluate their anticancer activity (Figure 6B). The cell survival rate of Au@DOX/CQ@TPP-DCD group with the DOX concentration of 25.6 μg/mL was 26.61% without laser irradiation, while the cell survival rate was 15.43% with NIR irradiation (808 nm, 1 W/cm^2^, 3 min). The photothermal effect of AuNPs under NIR irradiation together with mitochondria targeting chemotherapeutic DOX and autophagy inhibition exhibited excellent anticancer activity *in vitro*.

In Figure 6C and D, the concentration of CQ was changed to investigate the NIR irradiation effects on *in vitro* anticancer activity. As shown in Figure 6D, when the concentration of CQ was 60 μg/mL with NIR irradiation, the cell survival rate of Au@DOX/CQ@TPP-DCD was 28.22%, which showed the strongest inhibitory effect on 4T1 cells. The IC_50_ of Au@DOX/CQ@TPP-DCD with or without NIR irradiation were 6.98 and 5.85 μg/mL, respectively (Figure S12).

The cellular uptake of the drug-loaded nanoparticles was evaluated. As shown in Figure 6E, the fluorescence signals of 4T1 cells incubated with nanoparticles were observed by confocal laser scanning microscopy (CLSM) (Ex = 488 nm). The results showed that the drug-loaded nanoparticles began to enter the cells at 2 h, and the fluorescence intensity was weak at this time. The average fluorescence intensity of free DOX was 1.52, 1.29 and 1.19 times higher than that of the three drug-loaded nanoparticle groups of Au@DOX@TPP-DCD, Au@DOX/CQ@DCD and Au@DOX/CQ@TPP-DCD (Figure 6F). It implied that free DOX diffused quickly into cells [60]. The cellular internalization of nanoparticles occurred via endocytosis, which was slower than diffusion. At 5 h, strong green fluorescence was observed in all groups; the fluorescence intensity of Au@DOX/CQ@TPP-DCD was twice higher than that at 2 h, and the fluorescence range was enlarged, implying large amount of drug-loaded nanoparticles in 4T1 cells.

### Mitochondria targeting

**Figure 7.**
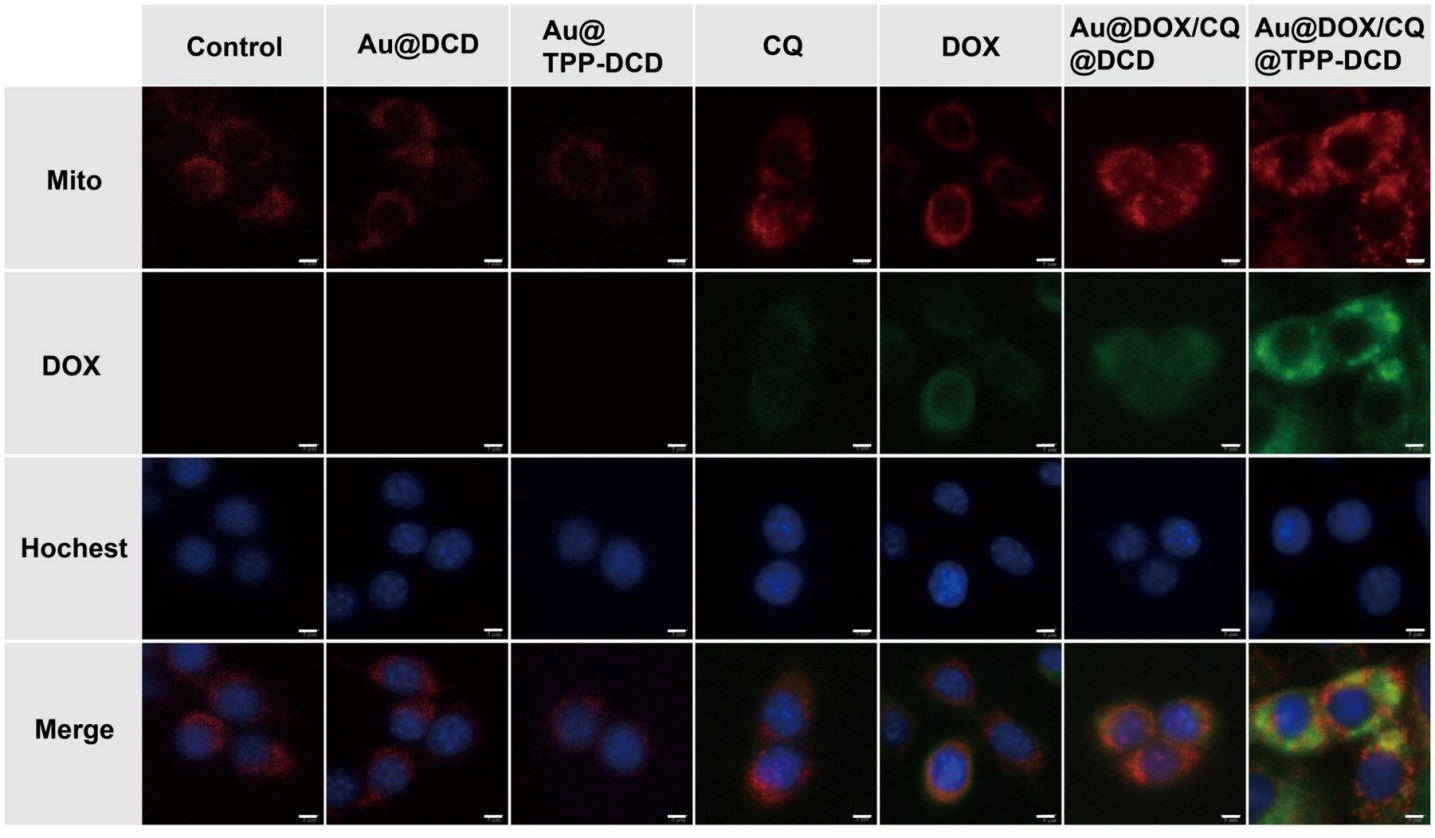
Mitochondrial targeting evaluation by confocal laser scanning microscopy images of 4T1 cells incubated with Au@DCD, Au@TPP-DCD, CQ, DOX, Au@DOX/CQ@DCD and Au@DOX/CQ@TPP-DCD. After different material groups (80 μg/mL) were co-incubated with 4T1 cells for 5 h, mitochondria and nuclei were stained with Mito-Tracke (red) and Hochest (blue), respectively. Red channel: λ_ex_=622 nm, λ_em_=648 nm; blue channel: λ_ex_=350 nm, λ_em_=461 nm (scale bar = 5 μm).

TPP is preferentially accumulated in mitochondria through endocytosis [43]. The co-localization of the two Au@DOX/CQ@DCD and Au@DOX/CQ@TPP-DCD nanoparticles was examined by CLSM. Mito-Tracker Deep Red (red fluorescence) was used as a mitochondrial dye; the blue fluorescence was derived from the nucleus stained by Hochest and the green fluorescence was marked for the fluorescence emitted by DOX excited at 488 nm. The Au@DCD, Au@TPP-DCD, CQ, DOX, Au@DOXCQ@DCD and Au@DOXCQ@TPP-DCD were co-cultured with 4T1 cells for 5 h; the CLSM observation is presented in Figure 7. Weak green fluorescence (marked as DOX) was observed in mitochondria for the free DOX and Au@DOX/CQ@DCD groups, indicating that both groups had minimal mitochondria-targeting capability. Obvious green fluorescence (i.e. DOX) overlap within the mitochondrial range (red fluorescence) was observed in the Au@DOX/CQ@TPP-DCD group, revealing efficient mitochondria-targeting of TPP.

### Evaluation of ROS

**Figure 8.**
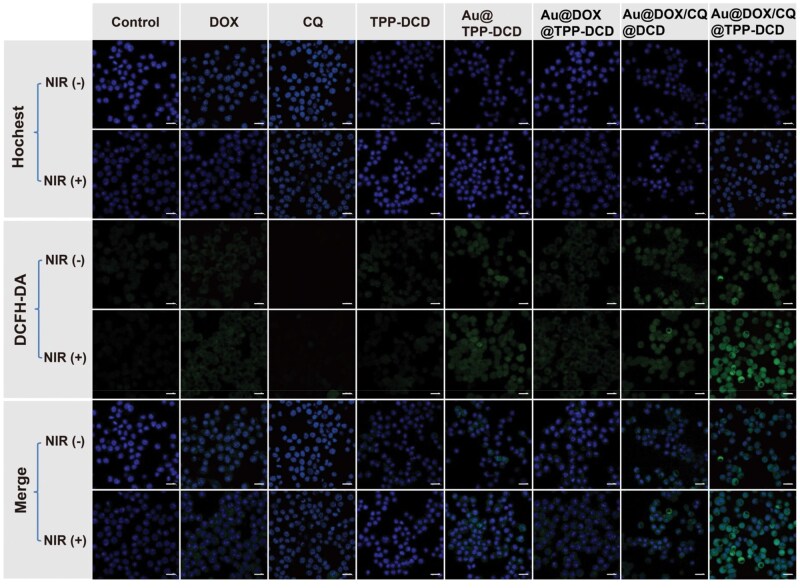
Evaluation of reactive oxygen species by confocal laser scanning microscopy images of 4T1 cells incubated with DOX, CQ, TPP-DCD, Au@TPP-DCD, Au@DCD, Au@DOX@TPP-DCD, Au@DOX/CQ@DCD and Au@DOX/CQ@TPP-DCD. The nanomaterials (80 μg/mL) were incubated with 4T1 cells for 5 h and with laser irradiation (808 nm, 1.0 W/cm^2^) for 3 min. The ROS and nucleus were stained with DCFH-DA (green) and Hochest (blue), respectively. Green channel: λ_ex_=488 nm, λ_em_=525 nm; blue channel: λ_ex_=350 nm, λ_em_=461 nm. (scale bar = 20 μm).

The intracellular ROS generation was detected by the ROS assay kit of DCFH-DA. DCFH-DA itself is non-fluorescent but can be hydrolyzed into DCFH by intracellular esterases. The DCFH molecule could be oxidized by ROS to DCF, emitting strong green fluorescence to be used as an indicator of ROS generation [61]. As shown in Figure 8, the control group showed no green fluorescence under NIR irradiation (808 nm, 1.0 W/cm^2^, 3 min), which proved that the NIR alone could not produce ROS. Nanoparticles with AuNPs as cores all exhibited green fluorescence under NIR irradiation. The green fluorescence in the 4T1 cells treated with Au@DOX/CQ@TPP-DCD under NIR irradiation was stronger than that of Au@DOX/CQ@DCD; it clearly demonstrated the mitochondria targeting mediated by TPP. The Au@DOX/CQ@TPP-DCD nanoparticles could promote the generation of ROS to enhance the therapeutic effect.

### Autophagy of cancer cells

**Figure 9.**
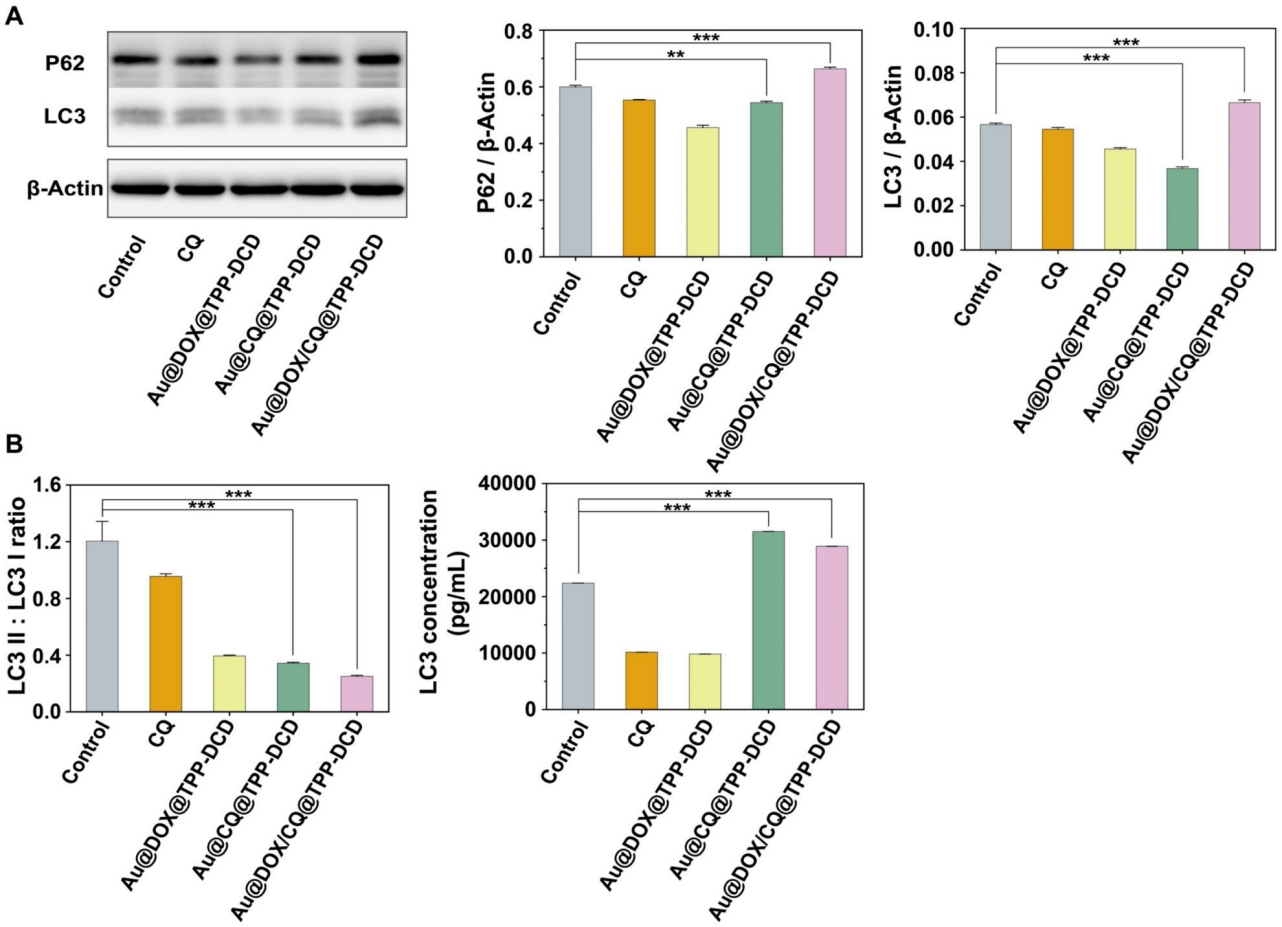
(**A**) Western blotting analysis of LC3 and P62 in the 4T1 cells treated with CQ, Au@DOX@TPP-DCD, Au@CQ@TPP-DCD, Au@DOX/CQ@TPP-DCD for 24 h. (**B**) ELISA analysis of LC3 in the 4T1 cells treated with CQ, Au@DOX@TPP-DCD, Au@CQ@TPP-DCD, Au@DOX/CQ@TPP-DCD for 24 h. The concentration of CQ was 30 μg/mL.

Both apoptosis and autophagy could induce cell death. Autophagy is a process of cell self-degradation, which plays an important role in adapting to metabolic stress, maintaining genome integrity and internal environment stability. Autophagy prevents apoptosis induced by antitumor drugs and further promotes drug resistance [62]. Therefore, the inhibition of autophagy is helpful to promote anticancer activity of drugs. The autophagy inhibitor CQ was encapsulated in the DOX-loaded nanoparticles to enhance the anticancer activity in this study. ELISA and western blotting techniques were used to detect the cellular expression levels of LC3-II/I and p62 proteins. As shown in western blotting results (Figure 9A), compared to the free CQ and Au@DOX@TPP-DCD groups, Au@DOX/CQ@TPP-DCD nanoparticles exhibited significant accumulation (or expression) of P62 in cells. P62 is a multidomain protein and an autophagy receptor, serving as a selective substrate for autophagy. It interacts with LC3 to form autophagosomes, and its degradation depends on autophagy. Therefore, the expression level of P62 is negatively correlated to autophagy activity [63]. The results showed that the effect of DOX/CQ co-loaded nanoparticles on tumor inhibition was better than that of single DOX-loaded nanoparticles, as CQ played an autophagic inhibitory role to increase the pH of the lysosome and inactivate the acid hydrolase in the lysosome, thus leading autophagic lysosome reformation to inhibit autophagy [63]. ELISA’s results showed that the expression of LC3 in cells increased and the LC3-II/I ratio decreased (Figure 9B) in the groups of Au@CQ@TPP-DCD and Au@DOX/CQ@TPP-DCD. Autophagy is a beneficial process in which intracellular lysosomes degrade misfolded proteins and damage or age organelles to maintain various cellular functional homeostasis. During the formation of autophagy, cytoplasmic type LC3 (LC3-I) hydrolyzes a small peptide and transforms into membrane type (LC3-II), which fuses with lysosomes to form autophagosomes [64]. Therefore, the magnitude of the LC3-II/I ratio means the high level of autophagy. When autophagy is inhibited, the LC3-II/I ratio will decrease and the expression level of LC3 will increase.

### *In vivo* distribution and imaging

The *in vivo* studies of the drug-loaded nanoparticles were carried out on 4T1 tumor-bearing BALB/c mice. The nanoparticles were labeled with fluorescent dyes (Cyanine 7, Cy7) to investigate the biological distribution *in vivo*. The treatment groups were free Cy7, free CQ, Au@DOX@TPP-DCD, Au@CQ@TPP-DCD, Au@DOX/CQ@DCD and Au@DOX/CQ@TPP-DCD.

**Figure 10.**
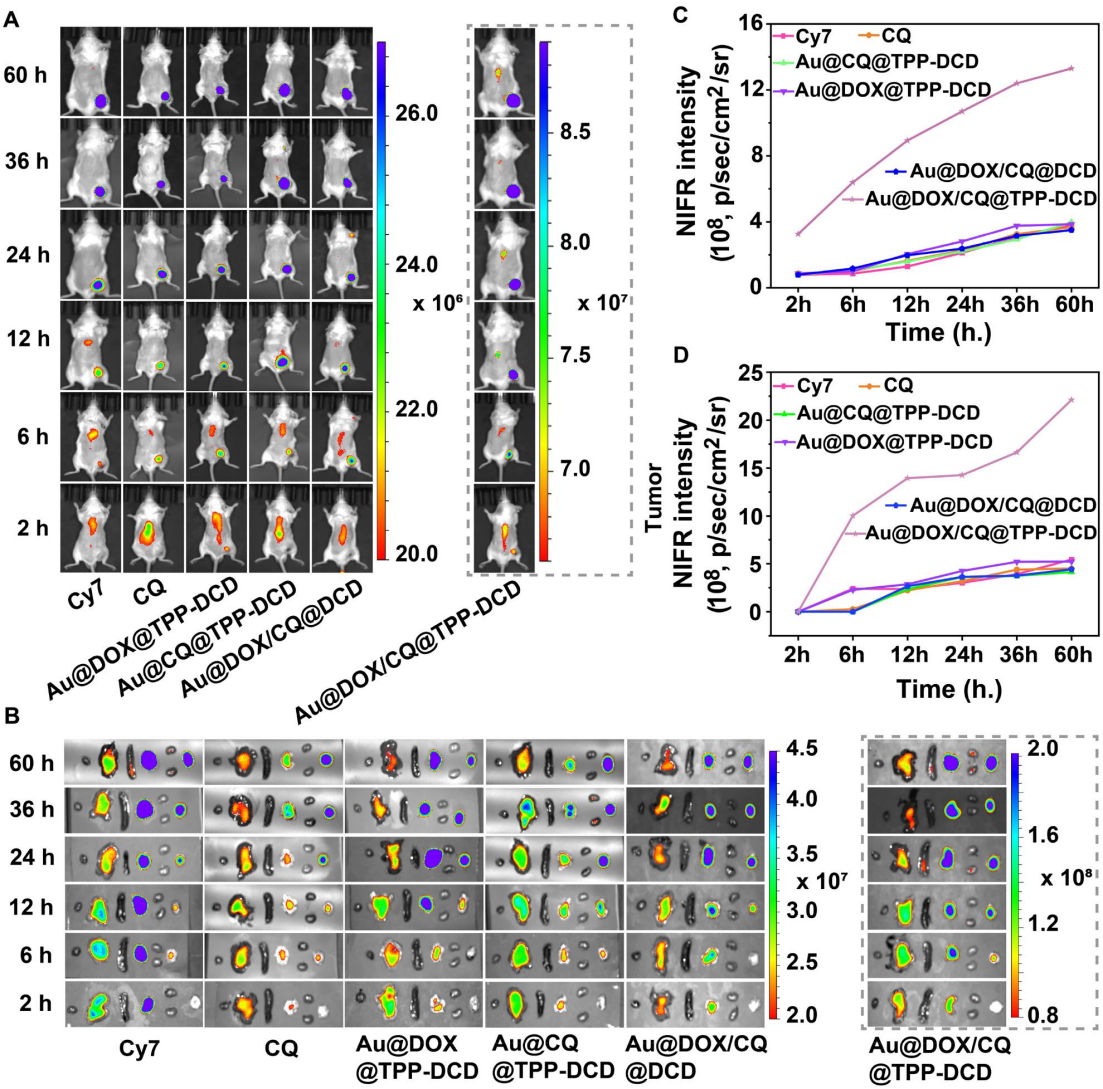
*In vivo* distribution and imaging study. Real-time fluorescence images (**A**) for 4T1-tumor-bearing BALB/c mice, and the matched fluorescence intensity (**C**). *Ex vivo* fluorescence images (**B**) for excised major organs and tumors (the sequences of isolated organs from left to right were heart, liver, spleen, lung, kidney and tumor tissue), and the matched average fluorescence intensity (**D**).

The drug-loaded nanoparticles were injected intravenously into tumor-bearing BALB/c mice at a DOX dose of 5 mg/kg. The mice were observed using an animal imaging system at 2, 6, 24, 36 and 60 h. As shown in Figure 10A, the fluorescence intensity of drug-loaded nanoparticles was strong in the livers of all the groups at 2 h. After 6 h, the fluorescence intensity in tumors with Au@DOX/CQ@TPP-DCD treatment was the most significant, indicating the aggregation of nanoparticles in tumors. At 12 h, in addition to the Au@DOX/CQ@TPP-DCD group, the nanoparticles of Au@DOX@TPP-DCD, Au@CQ@TPP-DCD and Au@DOX/CQ@DCD were gradually enriched in tumors. At 24 h, the nanoparticles in all treatment groups were enriched in the tumors, and kept fluorescence for as long as 60 h.

The images of the organs were used to confirm the tumor targeting of drug-loaded nanoparticles (Figure 10B). The fluorescence intensity of tumors in Au@DOX/CQ@TPP-DCD group was significantly higher than that of other groups. The fluorescence intensity in tumor-bearing Balb/c mice and major organs (Figure 10C and D) confirmed the efficient tumor targeting and accumulation of Au@DOX/CQ@TPP-DCD nanoparticles.

### *In vivo* anti-tumor effect

**Figure 11.**
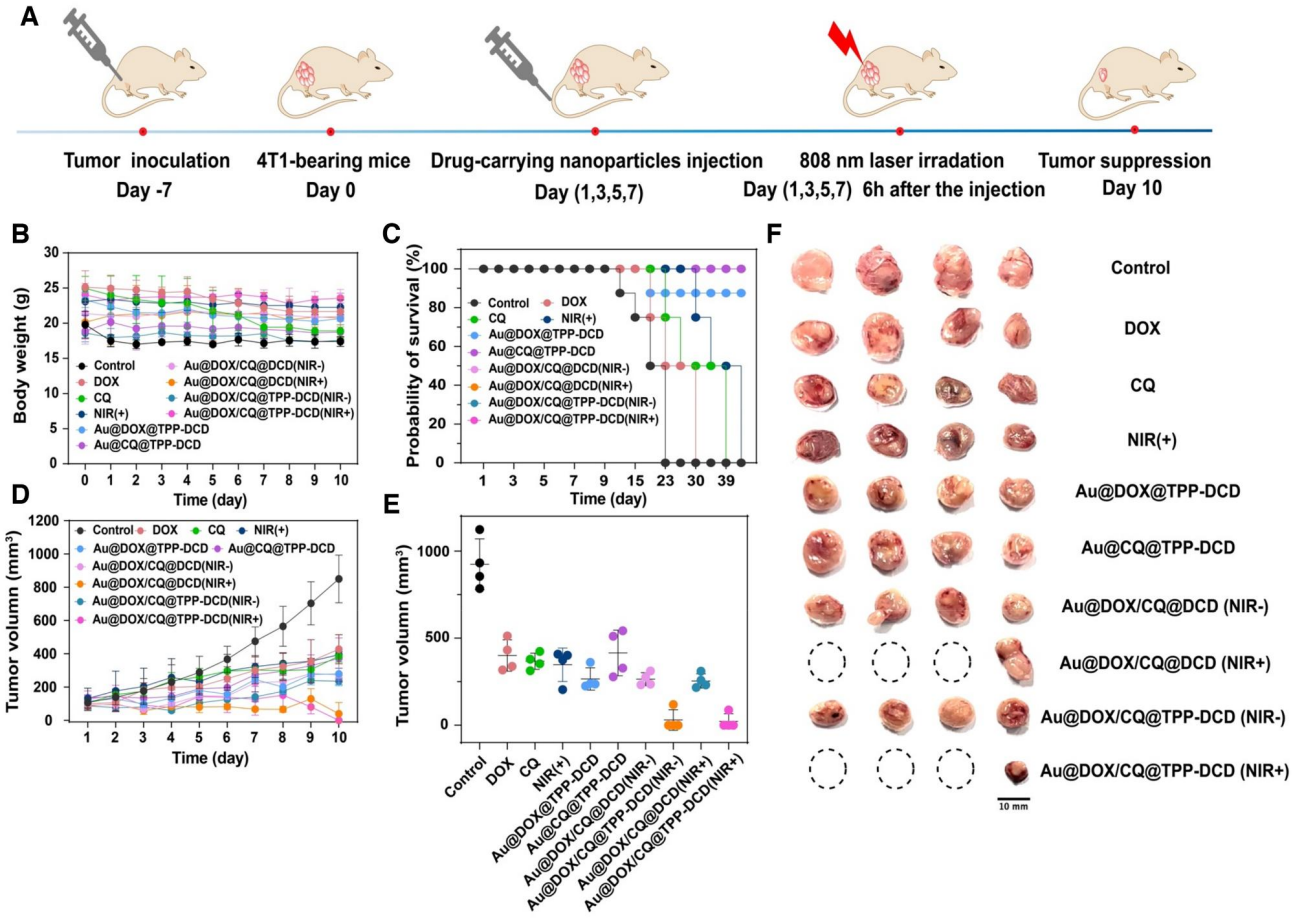
*In vivo* antitumor efficacy of different drug-loaded nanoparticles on the 4T1 tumor-bearing mice. (**A**) Schematic diagram of the tumor model establishment and treatment of different drug-carrying nanoparticles on the 4T1 tumor-bearing mice. (**B**) Evolution of body weights during the treatment. (**C**) Survival curve of 4T1 tumor-bearing mice after various treatments in 40 days. (**D**) Tumor growth curve after various treatments in 10 days. (**E**) Photos and (**F**) tumor volume of the dissected tumors obtained after 10 day therapy. The dashed areas in the (F) indicate that the tumor has been ablated. Ten treatment groups were (i) saline, (ii) free DOX, (iii) free CQ, (iv) saline with laser irradiation (808 nm), (v) Au@DOX@TPP-DCD, (vi) Au@CQ@TPP-DCD, (vii) Au@DOX/CQ@DCD, (viii) Au@DOX/CQ@DCD with laser irradiation (808 nm), (ix) Au@DOX/CQ@TPP-DCD, (x) Au@DOX/CQ@TPP-DCD with laser irradiation (808 nm).

All animal experiments were performed in line with the animal experiments ethical committee for care and use of research animals. The tumor-bearing mice were injected intravenously via the tail vein with a DOX dose of 5 mg/kg body weight. All samples were injected once every 2 days, and the treatment was completed after four injections (Figure 11A). The weight of mice in each treatment group was stable (Figure 11B). Figure 11C shows the survival rates of mice in each group within 40 days. The saline group mice died firstly, and all mice died gradually within 23 days. The drug-loaded groups, including Au@CQ@TPP-DCD, Au@DOX/CQ@DCD (with or without NIR irradiation), Au@DOX/CQ@TPP-DCD (with or without NIR irradiation), survived 100% at 40 days, and the survival rate of Au@DOX@TPP-DCD group was 87.5% at 40 days.

In the saline group, the tumors grew very fast, and the mortality rate of mice was 50% after 20 days of tumor invasion. The average tumor volume was 924.15 mm^3^±146.31 in 10 days. The average tumor volumes of (ii) free DOX, (iii) free CQ and (iv) saline with laser irradiation (808 nm) were 399.95 mm^3^±195.14, 367.27 mm^3^±46.33, 374.37 mm^3^±96.32, respectively, indicating that only NIR irradiation and single free drug treatment could only slightly inhibit tumor growth. The Au@DOX@TPP-DCD and Au@CQ@TPP-DCD groups showed slow tumor growth; the effect was not as significant as the Au@DOX/CQ@TPP-DCD and Au@DOX/CQ@DCD groups with co-loaded drugs. The anti-tumor effect of Au@DOX/CQ@TPP-DCD group was better than that of Au@DOX/CQ@DCD group. Importantly, when both groups of co-loaded drug were combined with NIR irradiation (viii) Au@DOX/CQ@DCD and (x) Au@DOX/CQ@TPP-DCD with NIR laser irradiation (808 nm), the average tumor volumes were 29.5 mm^3^ (viii) and 21.79 mm^3^ (x), respectively. Moreover, some tumor tissues were eliminated completely in these two groups as shown in Figure 11D and F. The group (x) of Au@DOX/CQ@TPP-DCD with NIR laser irradiation (808 nm) showed the most effective tumor suppression (Figure 11E).

**Figure 12.**
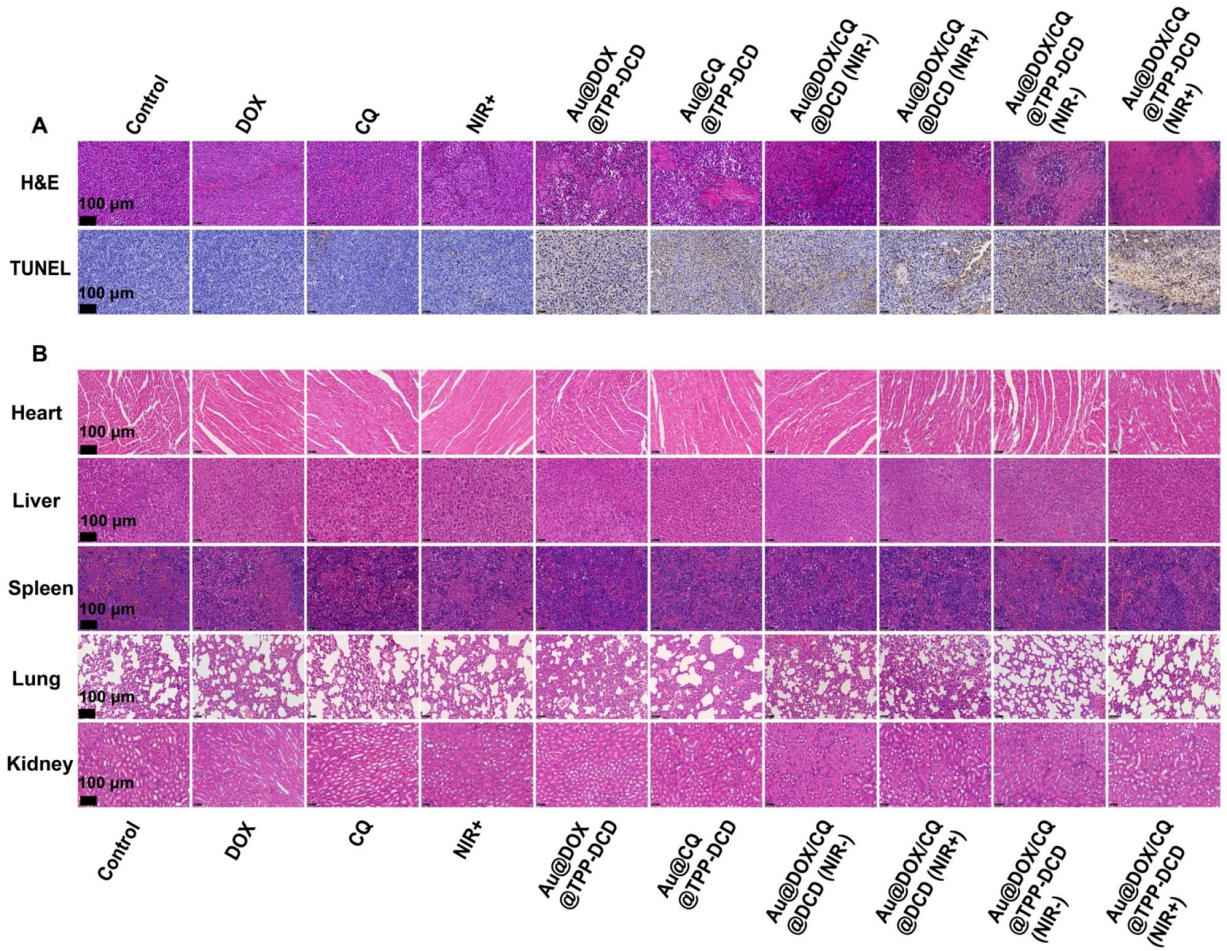
H&E and TUNEL staining. (**A**) H&E and TUNEL staining of tumor tissues excised from 4T1 tumor-bearing mice 10 days after different treatments. Scale bar: 50 μm. (**B**) H&E staining of heart, liver, spleen, lung and kidney from 4T1 tumor-bearing mice treated with different formulations: (i) control, (ii) free DOX, (iii) free CQ, (iv) only NIR irradiation, (v) Au@DOX@TPP-DCD, (vi) Au@CQ@TPP-DCD, (vii) Au@DOX/CQ@DCD, (viii) Au@DOX/CQ@DCD (NIR, 808 nm), (ix) Au@DOX/CQ@TPP-DCD and (x) Au@DOX/CQ@TPP-DCD (NIR, 808 nm). Scale bar: 50 µm.

To further illustrate the significance of Au@DOX/CQ@TPP-DCD with NIR irradiation, the excised tumor tissues were stained with H&E and TUNEL (Figure 12A). There was no significant change in the level of cell apoptosis in the (ii)–(iv) treatment groups. However, the apoptosis level of cells in the (v)–(x) treatment groups was significantly increased. Especially, the group (x) of Au@DOX/CQ@TPP-DCD with NIR laser irradiation (808 nm) showed the best significance in cell apoptosis and tumor suppression.

The organs of heart, liver, spleen, lung and kidney were dissected and stained with H&E. The results indicated that no obvious organ damage or inflammatory lesions were observed in the drug-loaded nanoparticles, indicating the negligible systemic toxicity of drug-loaded nanoparticles (Figure 12B).

In addition, blood and biochemical analyses were evaluated to investigate the side effects of drug-loaded nanoparticles. Hematology analysis showed that only the treatment groups of (viii) Au@DOX/CQ@DCD with laser irradiation (808 nm), and (x) Au@DOX/CQ@TPP-DCD with laser irradiation (808 nm) were normal, the white blood cell (WBC) count of both groups was within the normal reference range (Figure S13), while the white blood count of other treatment groups was significantly higher than the normal reference range, indicating the inflammatory response. However, all other important hematological indicators, including WBC, RBC, hemoglobin, mean corpuscular hemoglobin (MCH), mean corpuscular volume and mean corpuscular hemoglobin concentration (MCHC), were normal.

Blood biochemical analysis (Figure S14) showed that only two parameters related to liver function (aspartate aminotransferase (ALT) and alanine aminotransferase (AST)) in the free DOX treatment group were far beyond the normal reference range, indicating that free DOX could cause liver damage. The other treatment groups had random and slight fluctuations in the values of major organ biochemical markers such as liver and kidney (blood urea nitrogen and creatinine), but they remained within the normal reference range (Table S2). Particularly, the group (x) of Au@DOX/CQ@TPP-DCD with laser irradiation (808 nm) not only possessed better tumor inhibitory effect but also exhibited better blood biochemical indicators.

## Conclusion

The nanomedicines with PTT, autophagy inhibitor and mitochondrial targeting were constructed to enhance cancer therapy. The AuNPs cores were coated with (3-carboxypropyl) triphenyl phosphorus bromide and β-CD modified dextran to load chemotherapeutic DOX and the autophagy inhibitor CQ. The co-loaded DOX and CQ nanomedicines targeted cellular mitochondria and induced cell apoptosis and tumor suppression via chemotherapy and photothermal effect under NIR irradiation. The autophagy inhibition of the nanomedicines contributed greatly to the efficient synergistic therapy. The nanomedicine with mitochondria-targeting and autophagy inhibition is promising for the synergistic photothermal-chemotherapy of cancers.

## Supplementary Material

rbae141_Supplementary_Data
