# Supplementary material for: Mitochondria-targeting nanomedicines with autophagy inhibitor to enhance cancer photothermal-chemotherapy
Source: Regen Biomater. 2025 Jan 6;12:rbae141. doi: 10.1093/rb/rbae141 (PMC11925499; doi:10.1093/rb/rbae141)
Supplement: rbae141_Supplementary_Data [file rbae141_supplementary_data.docx]

Supporting Information

Mitochondria-targeting Nanomedicines with Autophagy Inhibitor to Enhance Cancer Photothermal-Chemotherapy

Shuqi Chen,^1^ Wenxia Gao,^1*^ Shuhua Chang,^2^ Bin He,^2*^ Congbo Zhang,^3^ Miaochang Liu,^3^ Xueting Ye^4*^

^1^ School of Pharmacy, Chengdu University, Chengdu 610106, China

^2^ National Engineering Research Center for Biomaterials, College of Biomedical Engineering, Sichuan University, Chengdu 610064 China

^3^ College of Chemistry and Materials Engineering, Wenzhou University, Wenzhou 325027, China

^4^ Department of Urology, the First Affiliated Hospital of Wenzhou Medical University, Wenzhou 325000, China.

* To whom correspondence should be addressed, E-mail: gaowenxia@cdu.edu.cn (W. Gao); [bhe@scu.edu.cn](mailto:bhe@scu.edu.cn) (B. He); [yexueting@wmu.edu.cn](mailto:yexueting@wmu.edu.cn) (X. Ye)

1. Experimental section

1. 1 Materials and methods

1.1.1 Materials

*β*-Cyclodextrin and monochloroacetic acid was purchased from Sinopharm Chemical Reagent. 1-ethyl-(3-dimethyllaminopropyl) carbodiiehydrochlide (EDC·HCl), 1-hydroxy-benzotriazole monohydrate (HOBT), *N, N*-diisopropylethylamine (DIEA), and 2-aminoethanethiol were obtained from Sigma-Aldrich Co. Ltd. Dextran 70 and (3-carboxypropyl) triphenylphosphonium bromide (TPP), ascorbic acid, cetyltrimethylammonium bromide (CTAB), HAuCl_4_·3H_2_O, and NaBH_4_ were purchased from Shanghai Macklin Biochemical Technology Co. Ltd. Doxorubicin hydrochloride (DOX·HCl, Zhejiang Hisun Pharmaceutical, China) was deprotonated according to the method previously reported.^1^ All the solvents were purchased from Energy-Chemical Co. Ltd. (China). Dulbecco’s modified Eagle’s medium (DMEM), RPMI 1640 medium, 100 x penicillin, 100 x streptomycin and fetal bovine serum (FBS) were purchased from HyClone Inc. and used for cytotoxicity test. Thiazolyl Blue Tetrazolium Bromide (MTT) were purchased from Shanghai Macklin Biochemical Technology Co. Ltd.

1.1.2 Characterizations

The ^1^H NMR spectra were performed on Bruker Avance II NMR spectrometer at 500 MHz using tetramethylsilane as the internal standard. The measurements of size and size distribution of the nanoparticles were carried out using a dynamic light scattering (DLS) spectrometer (Malvern Zetasizer Nano ZS). Scanning electron microscope (SEM, S4800, Hitachi Ltd, Tokyo, Japan) was employed to observe the morphology of the nanoparticles. The lyophilized nanoparticles were redispersed in distilled water and dropped onto silicon pellet, the samples were dried overnight at room temperature for SEM. Atomic force microscopy (AFM) analysis was conducted using a Dimension ICON AFM from Bruker Corporation in close contact mode. UV-vis absorption (Specord 200 PLUS) and Fluorescence spectra (F-7000, Hitachi, Japan) were used to measure the drug loading content, releasing profile and π-π interaction between drug and carriers.

XPS spectroscopy was conducted on a PHI-5702 multifunctional X-ray photoelectron spectrometer (Physical Electronics Inc., USA) to determine the chemical composition of the SAMs on Au surface, using Mg-Kα radiation as the exciting source. The binding energies of the target elements were determined at a pass energy of 29.35 eV, with a resolution of about ±0.3 eV. Electron binding energies were calibrated using the contaminated carbon (C1s: 285.0 eV)

1.2 Synthesis of mitochondrial targeting polymer TPP-DCD

1.2.1 Preparation of Carboxymethyl *β*-cyclodextrin (*β*-CD-COOH)

The monochloroacetic acid (4.725 g, 0.05mol) and sodium hydroxide (2 g, 0.05mol) were dissolved in 10 mL of deionized water, respectively. The two solutions were mixed, and *β*-CD (2.724 g, 0.0024 mol) was added. The mixture was stirred at 80 ^o^C for 20 min. Then sodium hydroxide was added dropwise to the mixture to keep the pH value of 8-10, and the mixture was stirred for 10 h at 80 ^o^C. The solution was cooled to room temperature and the pH value was adjusted to 4.0 with 6 M H_2_SO_4_. The solution was gradually dripped into the ethanol, and the precipitate was washed with ethanol and dried under vacuum overnight to obtain Compound 1 (carboxymethyl *β*-CD, *β*-CD-COOH).

1.2.2 Preparation of Compound 2 (*β*-CD-COOH-SH)

Compound 1 was further modified with 2-aminoethanethiol. Compound 1 (0.5675 g, 0.5 mmol) was dispersed in 7 mL deionized water, the mixture was heated and stirred until clarified. Then EDC (0.4793 g, 2.5 mmol) and HoBt (0.3378 g, 2.5 mmol) were added, the mixture was heated and kept at 80°C for 30min. 2-Aminoethanethiol (0.1929 g, 2.5 mmol) and DIEA (0.4 mL, 2.5 mmol) were added, the mixture was refluxed and stirred for 48 h. After reaction, the solution was cooled to room temperature, and gradually dripped into acetone to obtain the precipitate. The crude product was reconstituted in deionized water and then gradually dripped into acetone for precipitation. This process was repeated three times. The final product of Compound 2 (*β*-CD-COOH-SH) was obtained after vacuum drying.

1.2.3 Preparation of *β*-CD modified dextran (Compound 3, DCD)

Compound 2 (β-CD-COOH-SH) (0.2652 g, 1.2 mmol) was dissolved in 5 mL ultra-dried DMSO, EDC (0.4601 g, 2.4 mmol) and HOBT (0.3243g, 2.4mmol) were added in the solution. The mixture was heated to 70℃ and kept stirring for 1 h. Then Dextran (0.07g, 0.4 mmol) and DIEA (0.4 mL, 2.4 mmol) were added into the solution, and the mixture was stirred for 72h. The solution was cooled to room temperature, and gradually dripped into acetone to obtain the precipitation. The crude product was reconstituted in DMSO and then gradually dripped to acetone for precipitation. This process was repeated for three times. The final product of Compound 3 (DCD) was obtained after vacuum drying.

1.2.4 Preparation of (3-Carboxypropyl) triphenylphosphonium bromide modified dextran (Compound 4, Dex-TPP)

(3-Carboxypropyl) triphenylphosphonium bromide (TPP) (0.8586 g, 2 mmol) was dissolved in 7 mL ultra-dried DMSO, EDC (0.3834g, 2.0mmol) and HOBT (0.2702 g, 2.0 mmol) were added in the solution. The mixture was heated to 70℃ and kept stirring for 30min. Then Dextran 70 (0.28g, 4.0 *μ*mol) and DIEA (0.32 mL, 2.0 mmol) were added in the solution, and the mixture was stirred for 48h. After reaction, the solution was cooled to room temperature, and gradually dripped to acetone to obtain the precipitation. The crude product was reconstituted in DMSO and then gradually dripped to acetone for precipitation. This process was repeated for three times. The final product of Compound 4 (Dex-TPP) was obtained after vacuum drying.

1.2.5 Preparation of *β*-CD modified dextran (Compound 5, TPP-DCD)

Compound 2 (*β*-CD-COOH-SH) (0.2652 g, 1.2 mmol) was dissolved in 5 mL ultra-dried DMSO, EDC (0.4601 g, 2.4 mmol) and HOBT (0.3243 g, 2.4mmol) were added in the solution. The mixture was heated to 70℃ and kept stirring for 1 h. Then Compound 4 (Dex-TPP) (0.07 g, 0.4 mmol) and DIEA (0.4 mL, 2.4 mmol) were added in the solution, and the mixture was stirred for 72 h. After reaction, the solution was cooled to room temperature, and gradually dripped to acetone to obtain the precipitation. The crude product was reconstituted in DMSO and then gradually dripped to acetone for precipitation. This process was repeated for three times. The final product of Compound 5 (TPP-DCD) was obtained after vacuum drying.

1.3. Preparation of mitochondria-targeted nanoparticles

1.3.1 Preparation of Au nanoparticles (AuNPs)

AuNPs were prepared according to previous study.^2^ The seed-mediated growth method was employed to synthesize AuNPs. 3.7 mL of CTAB (0.1 M) were mixed with 150 *μ*L of HAuCl_4_ (0.01 M) and the volume was adjusted to 4.7 mL by adding distilled water. The ice-cold NaBH_4_ aqueous solution (0.01 M, 0.3 mL) was added to the mixture. CTAB coated Au nanoparticles were formed within 2-5 h. The growth solution for AuNPs was consisted with a mixture of 300 mL CTAB (0.1 M), 16 mL HAuCl_4_ (0.01 M), 3 mL AgNO_3_ (0.01 M), 6 mL H_2_SO_4_ (0.5 M), and 2.4 mL ascorbic acid (0.1 M). The growth was initiated by adding 1.4 mL of seeds and stopped after 12 h, the mixture was centrifuged twice at 6000rmp for 15 min. The synthesized AuNPs were further washed for another four times. The size and shape of the AuNPs were characterized by SEM.

1.3.2 Preparation of Au nanorods loaded nanoparticles

The AuNPs (1.6 mg/mL) was dispersed in 1 mL deionized water under [ultrasound](http://dict.youdao.com/w/ultrasound/). The compound 3 (20 mg) or compound 5 (20 mg) was dissolved in 2 mL deionized water, and the AuNRs dispersion was added dropwise to the solution. After stirred for 24 h, the mixture was centrifuged at 12000 × *g* for 10 min. The product was dialyzed in a dialysis tubing (Spectra/Por MWCO = 1000) and freeze-dried to obtain the Au nanorods of Au@DCD or Au@TPP-DCD.

1.3.3 Preparation of drug loaded nanoparticles

1.3.3.1 Preparation of drug loaded nanoparticles of Au@DOX@TPP-DCD

TPP-DCD (40 mg) was dispersed in deionized water (1.5 mL) and stirred for 4 h for dissolving completely. AuNPs (1.6 mg/mL) dispersion was added dropwise to the TPP-DCD solution under 1 min ultrasound, and NaCl solution (2.5 mol/L, 100 μL) was further added into the solution. The mixture was stirred at room temperature for 8 h to prepare the Au@TPP-DCD dispersion. Then, DOX (18 mg) was dissolved in 2 mL DMSO with ultrasound for 5 min, and slowly dropped into Au@TPP-DCD dispersion. The whole dropping process was carried out under ultrasound, the mixture was stirred at room temperature for another 12 h. Then the solution was dropped into 400 mL deionized water and stirred for 48 h, then centrifuged at 1,000 rpm for 2 min. The red precipitate at the bottom was discarded, and the upper solution was collected and lyophilized to obtain DOX-loaded nanoparticles of Au@DOX@TPP-DCD.

1.3.3.2 Preparation of drug loaded nanoparticles of Au@CQ@TPP-DCD

The TPP-DCD-Au dispersion was prepared as 1.3.2.

Chloroquine (CQ, 17.5 mg) was dissolved in 0.5 mL DMSO with ultrasound for 2 min, it was slowly dropped into Au@TPP-DCD dispersion. The whole dropping process was carried out under ultrasound, the mixture was stirred at room temperature for another 12h. Then the solution was dropped into 100 mL of deionized water, stirred for 48 h, then centrifuged at 1,000 rpm for 2 min. The upper solution was collected and lyophilized to obtain CQ-loaded nanoparticles of Au@CQ@TPP-DCD.

1.3.3.3 Preparation of drug loaded nanoparticles of Au@DOX/CQ@TPP-DCD

The TPP-DCD-Au dispersion was prepared as described in 1.3.2.

Chloroquine (CQ, 18.6 mg) was dissolved in 1.0 mL DMSO with ultrasound for 2 min, it was slowly dropped into Au@TPP-DCD dispersion and stirred for 30 min. DOX (22 mg) was dissolved in 2 mL DMSO with ultrasound for 5 min, dropped into the above mixture and stirred for 12 h. The solution was dropped into 600 mL deionized water, stirred for 48 h, then centrifuged at 1,000 rpm for 2 min. The upper solution was collected and lyophilized to obtain CQ-loaded nanoparticles of Au@DOX/CQ@TPP-DCD.

1.3.3.4 Preparation of drug loaded nanoparticles of Au@DOX/CQ@DCD

DCD (40 mg) was dispersed in deionized water (1.5 mL) and stirred for 4 h to make it fully dissolved. AuNPs (1.6 mg/mL) dispersion was added dropwise to the DCD solution under ultrasound, and NaCl solution (2.5 mol/L, 100 μL) was further added in the mixture. The mixture was stirred at room temperature for 8 h to prepare the Au@DCD dispersion.

Chloroquine (CQ, 18.6 mg) was dissolved in 1.0 mL DMSO with ultrasound for 2 min, which was slowly dropped into Au@DCD dispersion and stirred for 30 min. DOX (22 mg) was dissolved in 2 mL DMSO with ultrasound for 5 min, and dropped into the above mixture and stirred for 12h. The solution was dropped into 600 mL deionized water, stirred for 48 h, then centrifuged at 1,000 rpm for 2 min. The upper solution was collected and lyophilized to obtain CQ-loaded nanoparticles of Au@DOX/CQ@DCD.

1.3.3.5 The drug loading content (DLC) and encapsulation efficiency (EE)

The DOX-loaded nanoparticles (Au@DOX@TPP-DCD, Au@DOX/CQ@TPP-DCD, Au@DOX/CQ@DCD) were dispersed in DMSO with ultrasound for 5 min. The content of DOX was determined by fluorescence spectrophotometry in DMSO using calibration curve obtained from DOX/DMSO solutions with different DOX concentrations (A=41.35209C+57.98721, R^2^=0.9999, C (μg/mL)). The released DOX was determined by a fluorescence detector with excitation wavelength at 488 nm and emission wavelength at 594 nm.

The CQ loaded nanoparticles (Au@CQ@TPP-DCD, Au@DOX/CQ@TPP-DCD, Au@DOX/CQ@DCD) were dispersed in DMSO (1 mg/mL) with ultrasound for 5 min. A solution with 2.5 μg/mL concentration was then further prepared with deionized water containing trifluoroacetic acid. The content of CQ was determined by HPLC (Methanol : Acetonitrile : Water containing TFA (4:18:78, v/v), flow rate = 0.8 mL/min, UV = 343nm) in DMSO using calibration curve obtained from CQ/ H_2_O (containing TFA, 4mL/L) solutions with different CQ concentrations ( A=128518C+10684.66279, R^2^=0.9941, C (μg/mL)).

The whole procedure was in dark. The drug loading content (DLC) and encapsulation efficiency (EE) were calculated from the following formulae:

DLC (%) = (weight of drug in micelle/weight of drug loaded micelle) ×100%

EE (%) = (weight of drug in micelle/weight of drug in feeding) ×100%

1.4. Evaluation of photothermal performance

The AuNPs dispersion (Au concentration of 0.16 mg/mL) was placed in a sample bottle under dark and windless environment, it was irradiated under near-infrared light (NIR, 808nm) with different powers (0.5 W, 1.0 W, 1.5 W, 2.0 W, 2.5 W, 3.0 W) (MW-GX-808/10W，Shanghai Xilong Optoelectronics Technology Co., Ltd). All samples were kept in a fixed position with NIR illumination, and real-time temperature was recorded at different time via the thermal infrared imaging camera (FOTRIC 224S，Suzhou Nuofangke Precision Equipment Co., Ltd).

The cyclable photothermal effect of AuNPs was further explored. The AuNPs dispersion (Au concentration of 0.16 mg/mL) was irradiated under near-infrared light (NIR, 808 nm, 2.0 w), and the real-time temperature was recorded via infrared thermal imaging cameras at different time (0 min, 0.5 min, 1 min, 2 min, 3.5 min, 5.5 min, 8 min, and 15 min). After 15 min irradiation, the solution was cooled to room temperature, and the second irradiation was carried out. The above operation was repeated for six times. The temperatures were also recorded via the thermal infrared imaging camera.

1.5. In vitro release profiles

Drug-loaded nanoparticles (Au@DOX/CQ@TPP-DCD, Au@CQ@TPP-DCD) were dispersed in PBS (1 mL, ionic strength = 0.01 M, pH = 7.4). All the experiments were employed under sink conditions to ensure the good solubility of DOX. The mixture was put in dialysis tubings (Spectra/Por MWCO = 1000). The tubings were immersed in vials containing 20 mL of phosphate buffered saline (PBS, pH=6.0 or pH=7.4) solution, and put in a shaking bed at 37 °C. 1 mL of PBS solution was taken out and the same volume of PBS was added to the vials at prescribed time intervals. At [interval](http://dict.youdao.com/w/interval/) [time](http://dict.youdao.com/w/time/) of 0.5, 2, 4.5, 6.5, 11.5, 23.5, 35.5 and 62.5 h, the solution was exposed to NIR (808 nm, 2 W or 3 W) for 3 min. The released DOX was determined by a fluorescence detector with excitation wavelength at 484 nm and emission wavelength at 594 nm. The released CQ was determined by a HPLC (LC-20A, Shimadzu, Japan, mobile phase was water (with trifluoroacetic acid 4 mL/L): acetonitrile: methanol = 78:18:4, flow rate of 0.8 mL/min, UV = 343 nm). The release experiments were conducted in triplicate, and the results were demonstrated as mean ± SD.

1.6. In vitro cytotoxicity assessment

Conducting MTT experiments were used to explore the cytotoxicity of DCD and TPP-DCD, L929 and 4T1 cells were cultured in a 96-well plate for 12 hours. Subsequently, the materials were added for a total co-culture period of 24 hours with the material concentrations of 20, 40, 80, 160, 320, 640, and 1280 µg/mL. To the light exposure group, the sanples were co-cultured with cells for 12 hours of co-culture, then the samples were irradiated by 808nm (1 W) near-infrared laser for 3 minutes, followed by an additional 12 hours of incubation in the culture chamber. Serum-free culture medium containing 10% MTT was added, and the cells were co-cultured for another 4 hours. Finally, DMSO was used to dissolve the formazan, and the OD value at 490nm was detected using an microplate reader (Synergy H1, BIOTEK). Further investigation was carried out through live/dead staining (IX73, Olympus Corporation). Following the instructions of the reagent kit, L929 and 4T1 cells were co-cultured with DCD and TPP-DCD materials for 24 hours. Afterward, serum-free culture medium containing Calcein/PI dyes was added, and the cells were incubated in dark for 30 minutes. Observation was conducted using an inverted fluorescence microscope (IX73, Olympus Corporation) with the excitation wavelength of 488 nm.

In addition, the hemolysis assay was conducted to further demonstrate the biocompatibility of Au@DCD and Au@TPP-DCD. Au@DCD and Au@TPP-DCD with the concentrations of 80, 160, 320, and 640 µg/mL were co-incubated with post-treated rat red blood cell suspension for 2 hours. Physiological saline was used as the negative control, and deionized water served as the positive control. The supernatant was then collected, and the OD value at 542 nm was measured using an microplate reader (Synergy H1, BIOTEK). Hemolysis rate was calculated accordingly.

1.7 In vitro anticancer activity

The inhibition rates of different drug-loaded groups on 4T1 cells in vitro were explored through MTT experiments. 4T1 cells were seeded into a 96-well plate, after the cells were adhered, different drug-loaded nanoparticles were added with the measurement of DOX concentrations. The experimental groups included: DOX, Au@DCD, Au@TPP-DCD, Au@DOX@TPP-DCD, Au@DOX/CQ@DCD, and Au@DOX/CQ@TPP-DCD, with the DOX concentrations of 0.8, 1.6, 3.2, 6.4, 12.8, and 25.6 µg/mL. The concentrations were measured in terms of CQ for another set of experimental groups, the groups were CQ, Au@DCD, Au@TPP-DCD, Au@CQ@TPP-DCD, Au@DOX/CQ@DCD, and Au@DOX/CQ@TPP-DCD with the CQ concentrations of 1.875, 3.75, 7.5, 15, 30, and 60 µg/mL. The groups were further divided into light-exposed and non-light-exposed subgroups. In the light-exposed groups, after 5 hours of co-incubation, 808 nm (1 W) near-infrared laser irradiation was applied for 3 minutes, followed by continued incubation in the culture chamber for 19 hours. Consistent with section 2.6, the OD values at 490 nm were measured using an microplate reader (Synergy H1, BIOTEK).

1.8 Cellular uptake

5×10^4^ 4T1 cells were seeded in each well of a 24-well plate and cultured for 12 hours. After the cells adhered to the well surface, complete culture media containing DOX, Au@DOX@TPP-DCD, Au@DOX/CQ@DCD, and Au@DOX/CQ@TPP-DCD were added. After co-cultured for 2 and 5 hours, the culture medium was removed, and the wells were washed with sterile PBS for three times. Subsequently, an inverted fluorescence microscope (IX73, Olympus Corporation) was used, the fluorescence signals of the cells at an excitation wavelength of 488nm were observed.

1.9. Mitochondria targeting evaluation

5×10^4^ 4T1 cells were seeded in each well of a 24-well plate and cultured for 12 hours with cover slips placed in the wells. Once the cells adhered to the well surface, 1 mL of complete culture media containing Au@DCD, Au@TPP-DCD, CQ, DOX, Au@DOX/CQ@DCD, and Au@DOX/CQ@TPP-DCD were added. After co-cultured for 5 hours, the culture medium was removed, and the wells were washed with sterile PBS for three times. Subsequently, an equal concentration of Mito-Tracker Deep Red solution (Mito-Tracker Deep Red 633, a mitochondrial far-red fluorescent probe, Beyotime) was added, and the cells were incubated in the culture chamber for 20 minutes in dark. After washing with sterile PBS for three times, an equal concentration of Hochest dye (Beyotime) was added, and the cells were incubated for 5 minutes in the culture chamber. After gentlely washed with PBS twice, 800 µL of PBS was added. The cover slips were carefully removed using tweezers and placed upside down on a glass slide. Invertedly, the fluorescence signals of Mito and Hochest were detected using confocal laser scanning microscopy (CLSM, LSM880, Carl Zeiss AG) with excitation wavelengths of 622 nm and 350 nm, and emission wavelengths of 648 nm and 461 nm, respectively.

1.10 Evaluation of reactive oxygen species

A six-well plate was taken and cover slips were placed in each well. 500 µL of prepared poly-L-lysine solution was added to each well, ensuring even coverage of the cover slips. The poly-L-lysine solution was coated on the cover glass at room temperature for 1 hour. The solution was discarded and the glass was wash with sterile PBS for three times, the six-well plate was opened. The cover slips were air-dried in a clean bench. 4T1 cells were seeded into the six-well plate, 1.5 mL of cell suspension was added to each well to ensure the uniform dispersion of cells on the cover slips. The cells were incubated overnight in the culture chamber.

The culture medium was removed, and 1.5 mL of complete culture media containing DOX, CQ, TPP-DCD, Au@TPP-DCD, Au@DOX@TPP-DCD, Au@DOX@TPP-DCD, and Au@DOX/CQ@TPP-DCD were added. After co-cultured for 5 hours, the samples were irradiated with 808 nm laser for 3 minutes, then continuously incubated for 3 hours in the culture chamber. The culture medium was discarded, the cells were washed with sterile PBS for three times, pre-prepared DCFH-DA dye (Beyotime) was added with the same concentration. After incubated in dark for 20 minutes in the culture chamber, the cells were washed with sterile PBS for three times, the same concentration of Hochest dye (Beyotime) was added. After incubated for 5 minutes in the culture chamber, the cells were gently wash with PBS twice, 800 µL of PBS was added, the tweezers were used to remove the cover glass, it was placed upside down on a glass slide. Invertedly, the dual-channel fluorescence signals of DCFH-DA and Hochest were detected using confocal laser scanning microscopy (CLSM, LSM880, Carl Zeiss AG) at the excitation wavelengths of 488nm and 350nm, and the emission wavelengths of 525 nm and 461 nm, respectively.

1.11 Autophagy evaluation

4T1 cells were seeded into each well in a six-well plate with the addition of 1.5 mL of cell suspension to ensure uniform cell distribution. After the cells were adhered to the culture chamber, the culture medium was removed, 1.5 mL of complete culture media containing CQ, Au@DOX@TPP-DCD, Au@CQ@TPP-DCD, and Au@DOX/CQ@TPP-DCD were added. The concentration of CQ was set to 30 µg/mL. After co-cultured for 5 hours, the culture mediumwas discarded, the cells were washed with sterile PBS for three times, 500 µL of trypsin was added for digestion. The trypsin was discarded, the complete culture medium was used to resuspend the cells into a single-cell suspension. After centrifugated at 1000 rpm for 3 minutes, the cells precipitation was collected, the cell pellet was washed with pre-chilled PBS at 4°C, and repeatly washed for three times, the cells were resuspend in cold PBS containing PMSF for collection. After multiple freeze-thaw cycles for cell lysis using physical methods, the lysate was centrifuged with 10,000 rpm at 4°C for 10 minutes. The supernatant containing proteins was collected , aliquoted, and stored at -20°C.

The bicinchoninic acid (BCA) protein assay kit (Beyotime) was used to determine the protein concentration of each sample according to the instructions. The protein concentration of each sample was adjusted to be consistent. Following the instructions of the Elisa kit (Mouse Microtubule-associated proteins 1A/1B light chain 3B ELISA Kit #EK4552 SAB (Signalway antibody); Mouse Microtubule-associated proteins 1A/1B light chain 3A (MAP1LC3A) ELISA Kit #EK9937 SAB (Signalway antibody)) to detect changes in LC3 and LC3-II/I.

To further determine the changes in p62 protein content after treated with different materials, the analysis was conducted through Western blot experiments. Following the same steps as described above for adherent 4T1 cells, the culture medium was discarded, and the cells were washed with pre-chilled PBS. Total protein extraction solution was added, thoroughly pipetted, and placed in ice for 10-20 minutes. After centrifugated with 10000 rpm at 4℃for 10 minutes, the supernatant was collected. The bicinchoninic acid (BCA) protein assay kit (Beyotime) was used to detect and normalize the protein concentration of each sample.

The total protein was heated at 100℃ for 5 minutes, separated by SDS-PAGE gel electrophoresis, and transferred to a membrane through immunoblotting at a constant current of 300 mA for 60-90 minutes. The transferred membrane was blocked in 5% skimmed milk (TBST formulation), and the phosphorylation indicator was blocked in 5% BSA (TBST formulation) at 37℃ for 1 hour. Subsequently, the blocked membrane was washed with TBST twice, incubated overnight with the primary antibody at 4℃, washed with TBST for 5 times, and then incubated with the secondary antibody at room temperature on a shaker for 1 hour. After repeatedly washed with TBST for four times, the protein bands were visualized using an enhance chemiluminescence (ECL) kit and captured by a fluorescence chemiluminescence imaging system.

1.12. In vivo anti-tumor effect

Animal experiments comply with the National Regulations on the Administration of Laboratory Animals and were approved by the Animal Care and Use Committee of Sichuan University (Document no. 55, 2001). BALB/C mice (male, 18-20g, 5-6 weeks old) were purchased from Chengdu Dashuo Experimental Animals Co., Ltd. and raised in a specific-pathogen-free (SPF) environment. After depilation, 4T1 cells were subcutaneously injected into the right flanks of mice at a density of 1×10^7^ to establish a subcutaneous tumor model. Tumor-bearing mice were randomly divided into ten groups of PBS, DOX (5 µg/g), CQ (4.35 µg/g), NIR(+), Au@DOX@TPP-DCD (DOX, 5 µg/g), Au@CQ@TPP-DCD (4.35 µg/g), Au@DOX/CQ@DCD (NIR-) (DOX, 5 µg/g), Au@DOX/CQ@DCD (NIR+) (DOX, 5 µg/g), Au@DOX/CQ@TPP-DCD (NIR-) (DOX, 5 µg/g), Au@DOX/CQ@TPP-DCD (DOX, 5 µg/g). The treatment was initiated when the tumor volume reached 100-200mm^3^. The treatment regimen was involved intravenous injection every 24 hours with an injection volume of 200 microliters. The light-exposed groups received 808nm laser treatment (1.5 W/cm^2^, 6min) after each treatment for 6 hours, while the control group was treated with physiological saline. The tumor volume was calculated using the formula: tumor volume = (length × width^2^) / 2 and recorded. Simultaneously, to assess systemic toxicity of the treatment, mouse body weight was measured and recorded daily. After 10 days, the mice in all groups were randomly picked out and sacrificed.

After completing the five treatment sessions, mice were euthanized, and whole blood was collected from each group for hematological and biochemical analysis. Hematological analysis included white blood cells (WBC), red blood cells (RBC), hemoglobin (HGB), hematocrit (HCT), mean corpuscular volume (MCV), mean corpuscular hemoglobin (MCH), mean corpuscular hemoglobin concentration (MCHC), red cell distribution width (RDW), platelet count (PLT), mean platelet volume (MPV), platelet distribution width (PDW), and plateletcrit (PCT). Biochemical analysis included alanine aminotransferase (ALT), aspartate aminotransferase (AST), urea (UREA), and creatinine (CREA). Tumor tissues and other major organs including heart, liver, spleen, lungs, and kidneys were collected, fixed in 4% paraformaldehyde, dehydrated, embedded, sectioned for H&E analysis, and subjected to TUNEL analysis for tumor tissues. Tumor and organ slices were observed using a W10 slide scanner (Wisleap, WS-10, China).

1.13 In vivo distribution and imaging

The 4T1 tumor-bearing mice were randomly divided into 6 groups, with each group comprising 15 mice. In order to investigate the in vivo distribution of free Cy7, CQ, Au@DOX@TPP-DCD, Au@CQ@TPP-DCD, Au@DOX/CQ@DCD, and Au@DOX/CQ@TPP-DCD after intravenous injection, whole-body imaging of the mice was conducted at 2 h, 6 h, 12 h, 24 h, 36 h, and 60 h post-administration using the IVIS® Spectrum system (PerkinElmer). After euthanizing the mice by cervical dislocation, heart, liver, spleen, lungs, kidneys, and tumor tissues were collected. Ex vivo imaging was performed on these tissues using the imaging system to observe the distribution and fluorescence intensity of Cy7.

1.14 Statistics.

t-Test was used to determine the statistical significant differences. The probability value (P) of ≥ 0.05 means no significant (ns) difference between two groups; * P < 0.05, ** P < 0.01, and *** P < 0.001.

Fig. S1 ^1^H NMR spectrum of β-CD.

Fig. S2 ^1^H NMR spectrum of Compound 1 (*β*-CD-COOH).

Fig.S3 The FT-IR spectra of β-CD and β-CD-COOH-SH.

Fig. S4 ^1^H NMR spectrum of Dextran.

Fig.S5 The FT-IR spectra of DCD and TPP-DCD.

Fig.S6 The FT-IR spectra of Dextran and Dex-TPP.

Fig. S7 TGA images of β-CD and β-CD-COOH-SH.

Fig. S8 TGA images of DCD and TPP-DCD.

Fig. S9 Zeta potentials of nanoparticles.

Table S1. DLC and DLE of four drug-loaded nanoparticles.

|  | Au@DOX@  TPP-DCD | Au@CQ@  TPP-DCD | Au@DOX/CQ@  TPP-DCD | Au@DOX/CQ@  DCD |
| --- | --- | --- | --- | --- |
| DLC（%） | DOX：30.02% | CQ：29.46% | DOX：26.76%  CQ：22.63% | DOX：29.10%  CQ：21.38% |
| EE  （%） | DOX：7% | CQ：4% | DOX：5%  CQ：10% | DOX：3%  CQ：7% |

Fig. S10 Photothermal images of AuNPs with different concentrations under the same power NIR irradiation (808 nm, 2 W).

Fig. S11 Cyclic photothermal images of AuNPs and TPP-DCD-Au@DOX-CQ under NIR irradiation (808 nm, 2 W)

Fig. S12 (A) IC_50_ values for different groups, measured as DOX. (B) IC_50_ values for different groups, measured as CQ.

Fig. S13 The analysis results of hematology related indicators.

Fig. S14 Blood biochemistry data including liver function markers: ALT, AST, and kidney function markers: BUN, CR.

Table S2. Reference range of hematology-related indicators in normal mice

References

(1) Xu, J.; Zhu, X.; Qiu, L. Polyphosphazene vesicles for co-delivery of doxorubicin and chloroquine with enhanced anticancer efficacy by drug resistance reversal. *International Journal of Pharmaceutics* 2016, *498* (1), 70-81. DOI: 10.1016/j.ijpharm.2015.12.003.

(2) Liao, J.; Shi, K.; Jia, Y.; Wu, Y.; Qian, Z. Gold nanorods and nanohydroxyapatite hybrid hydrogel for preventing bone tumor recurrence via postoperative photothermal therapy and bone regeneration promotion. *Bioactive Materials* 2021, *6* (8), 2221-2230. DOI: 10.1016/j.bioactmat.2021.01.006.
